# Prevalence and Predictors of Substance Use Disorder Due to Gabapentinoids in Patients With Chronic Non‐Cancer Pain: A Cross‐Sectional Study

**DOI:** 10.1002/hsr2.72888

**Published:** 2026-07-28

**Authors:** Neele Kufeld, Klaus Weckbecker, Eva Münster, Norbert Scherbaum, Michael Specka, Johannes Just

**Affiliations:** ^1^ Department of Human Medicine, General Practice I and Interprofessional Care, Institute of General Practice and Primary Care Witten/Herdecke University Witten Germany; ^2^ Department of Psychiatry and Psychotherapy, LVR‐University Hospital Essen, Medical Faculty University of Duisburg‐Essen Essen Germany

**Keywords:** chronic non cancer pain, Gabapentin, substance use disorder

## Abstract

**Background and Aims:**

The increase in the use of gabapentinoids in the treatment of patients with chronic non‐cancer pain (CNCP) has drawn attention to the possibility of substance use disorder (SUD) due to gabapentinoids. Systematic literature reviews have provided evidence of the risk of SUD due to gabapentinoids within substance‐using populations. Despite concerns, prescription rates of gabapentinoids continue to increase in many countries, such as the USA, the UK, and Germany. Gabapentinoids are frequently prescribed off‐label for chronic pain regardless of neuropathic pain diagnosis, increasing the risk for problematic use. A recent literature review suggests that there is a potential for SUDs in CNCP, but robust data focusing specifically on this population is lacking. The present study aims to address this by investigating SUD due to gabapentinoids in a cohort of patients with CNCP.

**Methods:**

This study employs an anonymous, self‐reported questionnaire‐based cross‐sectional design. It was conducted among CNCP patients in *n* = 11 general practitioner practices across two regions of Germany. A total of 93 adult patients with CNCPs who were treated with gabapentinoids were included in the final analysis. The questionnaire utilized was based on validated and established diagnostic instruments, including questions aligned with the DSM‐5.

**Results:**

The data revealed a prevalence of 26.9% (*n* = 25) for at least a mild SUD in the sample, including 5.4% with severe SUD. Logistic regression with sociodemographic, pain‐related, and psychological variables revealed that male sex was a statistically significant predictor of SUD due to gabapentoids (Exp(B) = 3.97, *p* < 0.05; CI: 1.40–11.26). This analysis is limited by sample size and self reported data.

**Conclusion:**

The results suggest a relevant risk for SUDs due to gabapentinoids in CNCP patients. Special care should be taken when prescribing gabapentinoids to patients with CNCP. Male sex as a predictor of SUD due to gabapentinoids should be further investigated.

**Abbreviations**
CNCP: chronic non‐cancer pain
DSM‐5: Diagnostic and Statistical Manual of Mental Disorders 5
SUD: substance use disorder

## 1 Background

Gabapentin and pregabalin bind to the α2‐δ subunit of voltage‐gated calcium channels on nociceptive neurons and thus reduce calcium influx and can therefore be a useful co‐analgesic in neuropathic pain conditions [1, 2]. The significant increase in the use of gabapentinoids (gabapentin, pregabalin) in the medical treatment of patients with CNCP has drawn attention to the possibility of SUD due to gabapentinoids [3, 4]. Several systematic literature reviews have provided evidence of the clinically relevant risk of SUD due to gabapentinoids within substance‐using populations at both the epidemiologic and individual case levels [5, 6, 7]. Despite this, prescription rates of gabapentinoids continue to rise in many countries, such as the USA, the UK, and Germany [4, 8, 9]. The observed increase in gabapentinoid prescriptions cannot be fully explained by either a rise in the number of approved indications or an increase in the prevalence of these conditions [4]. Gabapentin and pregabalin are the first‐line medicines for chronic neuropathic pain, and other indications including epilepsy or generalized anxiety disorder [2]. An analysis of prescribing patterns on the basis of secondary health data provides evidence that gabapentinoids are frequently prescribed for off‐label use in chronic pain, irrespective of a neuropathic pain diagnosis [9]. This is concerning, as gabapentinoids can have nonnegligible side effects such as sedation, dizziness, cognitive deficits and euphoria [4, 10, 11]. In addition to that researchers raise awareness for potential problematic use of gabapentinoids when it comes to off label uses [12].

To date, CNCP patients receiving prescribed gabapentinoids have been underrepresented in research. The potential of developing a SUD due to gabapentinoids has been investigated mainly in people who use drugs, particularly in individuals with an opioid use disorder (OUD). In these groups, more than half of the subjects reported having used gabapentinoids at least once during their lifetime. They cite the following as their motives: alleviating withdrawal symptoms; enhancing the effects of other psychotropic substances; and experiencing psychotropic effects of gabapentinoids themselves [13, 14]. OUD seems to be the greatest risk factor for gabapentin misuse [15]. Two studies reported that 12%–13% of urine tests of patients with known OUD tested positive for gabapentinoids without a medical indication [13, 16]. Researchers suggest that there is a potential for SUDs in CNCP patients and those with psychiatric disorders, but no study has yet been conducted [15]. Further research is urgently needed, especially as concomitant gabapentinoid therapy is associated with a significant increase in the risk of opioid‐related death in patients receiving opioid therapy [17].

The present study addresses this gap by quantifying the prevalence of SUD due to gabapentinoids in a sample of patients with CNCP and by exploring potential predictors within this population.

## 2 Methods

This paper is reported according to the STROBE guideline [18].

### 2.1 Study Design

A cross‐sectional, written and self‐reported questionnaire‐based study was conducted.

### 2.2 Setting and Participants

A patient survey was conducted over a 9‐month period, starting on the 1 of July 2021, in 11 primary care practices in Germany. Access to the practices was through the teaching and research practice network of the Chair of General Practice and Interprofessional Care. This study was conducted locally in Lower Saxony and North Rhine Westphalia.

The inclusion criterion was receiving gabapentin or pregabalin for the treatment of chronic pain. Another inclusion criterion was age 18 years or older. Patients receiving gabapentinoids for epilepsy, patients with an OUD receiving maintenance treatment, and patients with insufficient German knowledge to complete the questionnaire were excluded. Staff were trained in how to administer the survey in practice. Participation was voluntary. All patients signed an informed consent form. In the participating practices, patients who met the inclusion criteria were given a questionnaire by a staff member.

The questionnaire was anonymous and did not collect any personally identifiable information. Patients who chose not to participate were only asked to provide their age and sex voluntarily for nonresponder analysis. After completing the questionnaire, the participating patients dropped it directly into a sealed container. This ensured anonymity in the field, as no personal reference can be made. The sealed container in which the questionnaires were collected was sent by the practices to the Chair of General Practice and Interprofessional Care at the end of the survey period.

### 2.3 Variables

The questionnaire covers demographics, pain location, pain duration, medications, duration of use and criteria for substance use disorder (SUD) due to gabapentinoids (based on the *Diagnostic and Statistical Manual of Mental Disorders 5* [DSM‐5]) [19].

### 2.4 Data Analysis

Descriptive analyses were conducted to describe the sample and analyse the prevalence of SUD in this sample. Binary logistic regression was conducted to determine which factors influence the presence of SUD due to gabapentinoids. The blocks entered simultaneously included demographic variables, pain‐related variables (such as pain duration, pain localization, and intake of benzodiazepines or opioids), and psychological variables (such as potential psychotropic effects of gabapentinoids: relaxation, euphoria, and anxiety). The dependent variable was SUD, dichotomized into no SUD and at least mild SUD. A significance level (alpha) of 0.05 was chosen. Missing data were treated by imputation with values that, if anything, biased the effect towards the null. Specifically, when symptom data were missing, a value of zero was assigned, indicating the absence of that symptom, which likely biases the results toward the null hypothesis. The data were entered into a restricted‐access database. IBM SPSS 27th edition was used as a statistical program.

### 2.5 Ethics

The participants were provided with written information and provided informed consent before completing the questionnaire. The study was reviewed and approved by the Ethics Committee of the University of Witten/Herdecke (No. 246/2020).

## 3 Results

### 3.1 Participants

**Figure 1:**
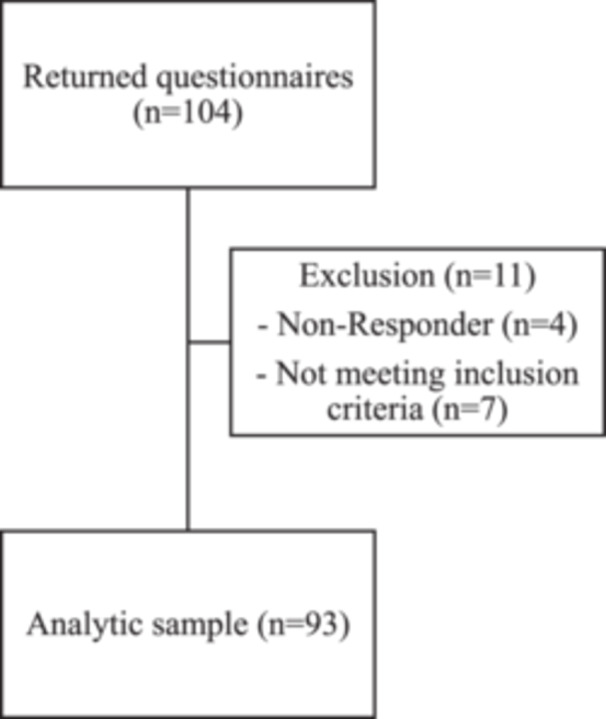
STROBE flow chart [18], documentation of the process from returned questionnaires to final analytic sample.

A total of 16 practices received the study materials and agreed to participate in the study. Out of these 11 practices returned questionnaires. Five other general practitioners received the material but did not return it. A total of 104 questionnaires were returned. Four of these were nonresponse surveys, meaning that only age and sex were completed. Of the 104 returned questionnaires, four were non‐response surveys and seven were excluded for not meeting inclusion criteria, resulting in a final analytic sample of 93 participants (Figure 1). These participants were not taking gabapentinoids for CNCP but were taking gabapentinoids for epilepsy or generalized anxiety disorder.

### 3.2 Descriptive Data

A total of 51.6% of the participants were female, and 48.4% were male. The mean age of the sample was 69 years (SD = 12.47), with most participants aged 43–65 years (40.9%). 25% of the participating patients were from small town areas, 32.7% were from middle town areas, and 42.3% were from large city areas.

Most participants had lower secondary school qualifications. The most common complaints were back pain (59.1%) and leg/foot pain (68.8%). A total of 68.8% reported more than two locations of CNCP. A total of 40.9% had experienced CNCP for more than 48 months. A total of 9.7% also took prescribed benzodiazepines. A total of 36.6% also took prescribed opioids (Table 1).

**Table 1:** Demographic characteristics of patients who received gabapentinoids for the treatment of CNCP.

Age	69 (SD = 12.47; range = 43–91)
Sex	Female: 51.6% (*n* = 48) Male: 48.4% (*n* = 45)
Education	No school: 5.4% (*n* = 5) Lower secondary school: 52.7% (*n* = 49) Intermediate secondary school: 17.2% (*n* = 16) University entrance qualification: 24.7% (*n* = 23)
Neuropathic pain	17.2% (*n* = 16)
Location of pain	Legs/feet: 68.8% (*n* = 64) Back: 59.1% (*n* = 55) Joints: 47.3% (*n* = 44) Hands/arms: 39.8% (*n* = 37) Neck: 35.5% (*n* = 33) Head: 19.4% (*n* = 18) Face: 7.5% (*n* = 7)
Multiple pain locations (≥ 2)	2 Locations of pain: 19.4% (*n* = 18) 3 Locations of pain: 16.1% (*n* = 15) 4 Locations of pain: 18.3% (*n* = 17) 5 Locations of pain: 7.5% (*n* = 7) 6 Locations of pain: 3.2% (*n* = 3) 7 Locations of pain: 4.3% (*n* = 4)
Duration of pain	More than 6 Months: 11.8% (*n* = 11) More than 12 Months: 4.3% (*n* = 4) More than 18 Months: 1.1% (*n* = 1) More than 24 Months: 38.7% (*n* = 36) More than 48 Months: 40.9% (*n* = 38)
Additional medications	Benzodiazepines: 9.7% (*n* = 9) Opioids: 36.6% (*n* = 34) Both: 6.5% (*n* = 6)

*Note: n* = 93.

### 3.3 Main Results

The prevalence of SUD (according to the DSM‐5 score [19]) due to gabapentinoids was 26.9% in the present sample of *n* = 93. Among these patients, 21.5% had mild‐to‐moderate SUD, and 5.4% had severe SUD (Table 2).

**Table 2:** Prevelence of SUD due to gabapentinoids in patients prescribed gabapentinoids for CNCP.

No SUD	73.1% (*n* = 68)
Mild SUD	21.5% (*n* = 20)
Moderate–Severe SUD	5.4% (*n* = 5)

*Note:* 0–1 symptoms = no SUD, 2–3 symptoms = mild, 4–5 symptoms = moderate, six or more symptoms = severe SUD. *n* = 93 [20].

Most commonly, patients reported that they were taking gabapentinoids for longer or longer than intended and that they wanted to reduce the dose (Table 3).

**Table 3:** Criteria of SUD in CNCP patients who received gabapentinoids for the treatment of CNCP.

Criteria	Applies	Does not apply
Intake longer/more than prescribed/intended	33.1% (*n* = 28)	69.9% (*n* = 65)
Persistemt desire or unsuccessful attempts to reduce or control use	35.5% (*n* = 33)	64.5% (*n* = 60)
Excessive time spent obtaining, using, or recovering from substance use	9.7% (*n* = 9)	90.3% (*n* = 84)
Craving or strong urge to use	5.4% (*n* = 5)	94.6% (*n* = 88)
Failure to meet major work, school, or home role obligations	4.3% (*n* = 4)	95.7% (*n* = 89)
Continued use despite social or interpersonal problems	1.1% (*n* = 1)	98.9% (*n* = 92)
Reduced or discontinuied importend social, occupational, or recreational activities	12.9% (*n* = 12)	87.1% (*n* = 81)
Recurrent use in physically hazardous situations	6.5% (*n* = 6)	93.6% (*n* = 87)
Continued use despite awareness of physical or psychological harm	16.2% (*n* = 15)	83.9% (*n* = 78)

*Note: n* = 93. Criteria in accordance with DSM‐5 [19].

#### 3.3.1 Logistic Regression

Only one of the calculated blocks yielded significant values; thus, only this block is reported below. The full regression analysis can be found in the Supporting Information (Supporting Information S1: Tables S1–10). The model as a whole is significant, with *p* = 0.03.

Logistic regression revealed a significant association between male sex and the presence of SUD (Exp(B) = 3.97, *p* < 0.05; CI: 1.40–11.26). In addition, there is a association of Exp(B) = 4.04 between not having a high school diploma and SUD compared with having a high school diploma (Table 4). This is not significant.

**Table 4:** Risk factors for diagnosis “substance use disorder” due to gabapentinoids in CNCP patients who received gabapentinoids for the treatment of CNCP.

	OR (95% CI)	*p*
Age	0.96 (0.29–3.16)	0.94
Gender	3.97 (1.40–11.26)	0.009
Education	4.04 (0.48–34.17)	0.20

*Note:* Logistic regression analysis, *n* = 93; significance of OR. Reference group in bold print—Age: **youngest** [43–65], middle [66–80], oldest [81–91]; gender: **male**, female; education: no school, lower secondary school, intermediate secondary school, **university entrance qualification**.

## 4 Discussion

### 4.1 Key Results

The prevalence of a substance use disorder caused by gabapentinoids was 26.9% in this sample of CNCP who receive prescribed gabapentinoids. Among the demographic, pain, and psychological variables included in the logistic regression, only sex (male) was significantly associated with having a higher risk of SUD. This risk factor is in accordance with literature on SUDs [21].

### 4.2 Limitations and Biases

As this questionnaire is based only on information provided by the patients themselves, there may be some bias. There was no link between the questionnaires and existing patient records in the practices, so the results are based on patients' answers concerning their reasons for taking gabapentinoid, the use of other medicines and sociodemographics. Potential bias variables, such as gender, were recorded so that adjustments could be made later in the analyses.

In terms of validity, questions have been raised regarding the clinical and epidemiological effects of the lowered diagnostic thresholds of the new DSM‐5 definition for SUD, which may lead to overdiagnosis of the condition. In the past, it has been theorized that a diagnosis of mild SUD, at most, points to a problematic pattern of substance use and the need for preventive measures. In contrast, moderate‐to‐severe SUD, with good agreement with ICD‐10 and DSM‐IV dependence diagnoses, might provide a good indication of a more severe disorder and a need for therapy addressing that issus [22]. The prevalence of 26.9% could be overestimated, as there are indications of false positive answers in the CNCP, especially for the most common positive symptoms [23].

Therefore, one could theorize that 5.4% of patients with moderate to severe SUD are high‐risk patients, whereas 21.5% with mild SUD do not suffer negative consequences but do experience some distress due to their gabapentinoid use and are in need of freuquent follow‐up visits. Other relevant limitations of this work include the localized approach. This study was conducted locally in Lower Saxony and North Rhine Westphalia, albeit two regions that cover appx. 20% of the German population. Only self‐reported data were available. Due to the self‐reported nature of the study design, this study lacks precise information regarding the specific medication (gabapentin or pregabalin), dose, dosing frequency, and duration of medication use. There are indications of possible diagnostic inaccuracies when paper‐based questionnaires are used to ascertain OUD criteria [24]. This limitation in diagnostic accuracy may also have occurred here. The decision to use self‐reports in this study was made primarily because linking the questionnaires to patient records would have required a more complex anonymization process. This would have been more difficult to implement in practices suffering from a high workload. It was also assumed that patient participation could be lower, as the topic of SUDs is associated with stigmatization. For the same reason, additional SUDs werde not assessed, which may represent a limitation of the study. The handling of missing values, for example by coding them as the absence of symptoms, may have introduced systematic bias in the estimates. However, within the exploratory design of this study, this approach was chosen to bias potential effects, if any, toward the null.

A key limitation of this study is the relatively small sample size, which may limit the generalizability of the findings and reduce the statistical power of the analysis. Thus, the results, particularly those of the logistic regression analysis, should primarily be interpreted as an exploratory foray into this research are. Participants were recruited from 11 different practices, which may have introduced some clustering effects. Although this was not accounted for analytically due to the sample size, the potential impact on the results is likely limited.

### 4.3 Interpretation

The prevalence of a SUD due to gabapentinoids was high, with approximately a quarter of the sample appearing to be affected (total 26.9%, moderate and severe: 5.4%). To our knowledge, no previous data on SUD due to gabapentinpids in patients prescribed gabapentinoids for CNCP have been published. Our findings show the prevalence was high, with a total of 26.9% meeting SUD criteria based on self‐report, with 5.4% being moderate to severe. This is consistent with a methodologically similar study in the same geographic area reporting a prevalence rate of 26.5% with OUD due to opioid pain medication [24].

The results suggest that special care should be taken when prescribing gabapentinoids to patients with chronic pain. An important clinical aspect is the strict application of indications, e.g., there should be a known or at least very likely neuropathic component of the pain or another indication, such as a comorbid anxiety disorder. In this context, the increase in off‐label prescriptions of gabapentinoids in Germany is particularly problematic [9]. It may be useful to monitor patients who receive gabapentinoids for CNCP for psychotropic side effects that limit their quality of life or may lead to SUD. If pain reduction cannot be achieved within 4–8 weeks of therapy, gabapentinoids should be discontinued gradually at a pace of 50–100 mg/week for pregabalin and 300 mg/4 days for gabapentin, which is necessary [25].

### 4.4 Generalizability

In terms of generalizability, limitations include that we only approached practices in two areas of Germany (Lower Saxony and North Rhine Westphalia) and were able to recruit only 11 out of 16 general practitioner practices which may constitute a selection bias. Age and sex were similar to comparable populations in Germany and other countries although the population seems to be slightly older than patients suffering from CNCP [9, 22, 24]. This suggests that a selection bias at a practices level is rather unlikely.

## 5 Conclusion

This study provides preliminary evidence that a substantial proportion of patients with CNCP who are prescribed gabapentinoids may meet DSM‐5 criteria for SUD, with prevalence estimates comparable to those observed for opioid‐related SUDs in similar populations. These findings highlight the necessity of careful risk–benefit assessment when prescribing gabapentinoids, particularly given the increasing off‐label use and the potential for psychotropic side effects that may contribute to misuse. While the generalizability of the findings is limited by the regional recruitment strategy, the self‐reported nature of the data, and the relatively small sample size, the observed patterns warrant further investigation. Further research is needed to better understand the development and clinical presentation of substance use disorders related to gabapentinoids in patients with CNCP. Many aspects of the addictive potential of gabapentinoids remain unclear. Further research is needed on the specific psychotropic (side) effects that make gabapentinoids a drug of interest for patients seeking to manipulate their mental state through the use of gabapentinoids as well as strategies for the treatment of SUD due to gabapentinoids, e.g., detoxification protocols.

## Author Contributions

**Neele Kufeld:** conceptualization, methodology, formal analysis, project administration, data curation, visualization, writing – original draft, investigation. **Klaus Weckbecker:** conceptualization, writing – review and editing, methodology, supervision. **Eva Münster:** conceptualization, methodology, writing – review and editing, formal analysis, supervision. **Norbert Scherbaum:** conceptualization, methodology, writing – review and editing, supervision. **Michael Specka:** methodology, conceptualization, writing – review and editing, formal analysis, supervision. **Johannes Just:** conceptualization, methodology, validation, writing – review and editing, supervision, formal analysis, project administration.

## Funding

The authors have nothing to report.

## Ethics Statement

The study was reviewed and approved by the Ethics Committee of the University of Witten/Herdecke (No. 246/2020).

## Consent

The participants were provided with written information and provided informed consent before completing the questionnaire.

## Conflicts of Interest

The authors declare no conflicts of interest.

## Transparency Statement

Neele Kufeld and Johannes Just affirm that this manuscript is an honest, accurate, and transparent account of the study being reported; that no important aspects of the study have been omitted; and that any discrepancies from the study as planned have been explained.

## Supporting information


Supporting File 1

Supporting File 2

## Data Availability

The data that support the findings of this study are available from the corresponding author upon reasonable request. Data available on request from the authors.
