# Supplementary material for: Prevalence and Predictors of Substance Use Disorder Due to Gabapentinoids in Patients With Chronic Non‐Cancer Pain: A Cross‐Sectional Study
Source: Health Sci Rep. 2026 Jul 28;9(8):e72888. doi: 10.1002/hsr2.72888 (PMC13412543; doi:10.1002/hsr2.72888)
Supplement: Supplementary file 1 — Supporting File 1 [file HSR2-9-e72888-s001.docx]

Supplementary Tables

Logistic Regression

Table 1. Case Processing Summary

| Unweighted cases | | N | % |
| --- | --- | --- | --- |
| Selected Cases | Includes in analysis | 93 | 100.0 |
|  | Missing cases | 0 | 0.0 |
|  | Total | 93 | 100.0 |
| Unselected cases | | 0 | 0.0 |
| Total | | 93 | 100.0 |

1. Once the weighting takes effect, you will find the total number of cases in the classification table.

Table 2. Coding of categorical Variables

|  | | Frequency | Parameter encoding | | | |
| --- | --- | --- | --- | --- | --- | --- |
|  |  |  | (1) | (2) | | (3) |
| Eduacational Attainment | None (1) | 5 | 1.000 | 0.000 | 0.000 | |
|  | Lower secondary school certificate (2) | 49 | 1.000 | 0.000 | 0.000 | |
|  | Intermediate secondary school certificate (3) | 16 | 0.000 | 0.000 | 1.000 | |
|  | University entrance qualification (4) | 23 | 0.000 | 0.000 | 0.000 | |
| Age | 43-65 years (1) | 40 | 0.000 | 0.000 |  | |
|  | 66-80 years (2) | 28 | 1.000 | 0.000 |  | |
|  | 81-91 years (3) | 25 | 0.000 | 1.000 |  | |
| Sex | Female (0) | 48 | 0.000 |  |  | |
|  | Male (1) | 45 | 1.000 |  |  | |

Table 3. Calssification table

| Observed | | Predicted | | |
| --- | --- | --- | --- | --- |
|  |  | Substance-use-disorder 1_0 | |  |
|  |  | 0 | 1 | Percentage of correct |
| Step 0 SUD yes/no | no | 40 | 0.000 | 100,0 |
|  | yes | 28 | 1.000 | 0.0 |
| Total Percentage | |  |  | 68.8 |

a. Costant included in the model

b. The cutoff value is ,500

Table 4. Variables in the equation

|  | Regression coefficient B | Standard error R | Wald | df | Sig. | Exp(B) |
| --- | --- | --- | --- | --- | --- | --- |
| Step 0 constant | -0.792 | 0.224 | 0.224 | 12.505 | <0.001 | 0.453 |

Table 5. Variables not in the equation

|  |  | Value | df | Sig. |
| --- | --- | --- | --- | --- |
| Step 0 variables | Age | 0.801 | 2 | 0.670 |
|  | Age (1) | 0.714 | 1 | 0.398 |
|  | Age (2) | 0.370 | 1 | 0.543 |
|  | Educational Attainment | 6.440 | 3 | 0.092 |
|  | Educational Attainment (1) | 2.045 | 1 | 0.153 |
|  | Educational Attainment (2) | 5.603 | 1 | 0.018 |
|  | Educational Attainment (3) | 1.422 | 1 | 0.233 |
|  | 1=m, 0=f(1) | 4.951 | 1 | 0.026 |
| Overall Statistics | | 13.446 | 6 | 0.036 |

Table 6. Omnibus-Test of the Model Coefficients

|  | | Chi-Quadrat | df | Sig. |
| --- | --- | --- | --- | --- |
| Step 1 | Step | 14.185 | 6 | 0.028 |
|  | Block | 14.185 | 6 | 0.028 |
|  | Model | 14.185 | 6 | 0.028 |

Table 7. Model Summary

| Step | -2 Log-Likelihood | Cox & Snell R-Quadrat | Nagelkerkes R-Quadrat |
| --- | --- | --- | --- |
| 1 | 101.238a | 0.141 | 0.199 |

a. Estimation stopped at iteration number 4 because the parameter estimators changed by less than .001

Table 8. Classification table a

| Observed | | Predicted | | |
| --- | --- | --- | --- | --- |
|  |  | Substance-use-disorder 1_0 | |  |
|  |  | 0 | 1 | Percentage of correct |
| Step 0 SUD yes/no | no | 54 | 10 | 84.4 |
|  | yes | 16 | 13 | 44.8 |
| Total Percentage | | 72.0 | | |

a. The cutoff value is 0.500

Table 9. Variables in the equation

|  |  | Regression coefficient B | Standard error | Wald | df |
| --- | --- | --- | --- | --- | --- |
| Step 1a | Age |  |  | 0.043 | 2 |
|  | Age(1) | -0.045 | 0.610 | 0.006 | 1 |
|  | Age (2) | 0.089 | 0.597 | 0.022 | 1 |
|  | Educational Attainment |  |  | 7.748 | 3 |
|  | Educational Attainment (1) | 1.396 | 1.089 | 1.644 | 1 |
|  | Educational Attainment (2) | -0.995 | 0.594 | 2.802 | 1 |
|  | Educational Attainment (3) | 0.442 | 0.714 | 0.384 | 1 |
|  | 1=m, 0=f(1) | 1.380 | 0.532 | 6.738 | 1 |
| Constant | | -1.241 | 0.627 | 3.920 | 1 |

Table 10. Variables in the equation

|  | | | | 95% confidence interval for EXP(B) | |
| --- | --- | --- | --- | --- | --- |
|  |  | Sig. | EXP(B) | Lower Limit | Upper Limit |
| Step 1a | Age | 0.979 |  |  |  |
|  | Age (1) | 0.941 | 0.956 | 0.289 | 3.159 |
|  | Age (2) | 0.881 | 1.094 | 0.340 | 3.522 |
|  | Educational Attainment | 0.052 |  |  |  |
|  | Educational Attainment (1) | 0.200 | 4.041 | 0.478 | 34.166 |
|  | Educational Attainment (2) | 0.094 | 0.370 | 0.115 | 1.185 |
|  | Educational Attainment (3) | 0.536 | 1.556 | 0.384 | 6.306 |
|  | 1=m, 0=f(1) | 0.009 | 3.974 | 1.402 | 11.264 |
| Constant | | 0.048 | 0.289 |  |  |

a. Variables entered in Step 1: Age, Educational Attainment, 1=m, 0=w.
