# Supplementary material for: Prevalence and Predictors of Substance Use Disorder Due to Gabapentinoids in Patients With Chronic Non‐Cancer Pain: A Cross‐Sectional Study
Source: Health Sci Rep. 2026 Jul 28;9(8):e72888. doi: 10.1002/hsr2.72888 (PMC13412543; doi:10.1002/hsr2.72888)
Supplement: Supplementary file 2 — Supporting File 2 [file HSR2-9-e72888-s002.pdf]

Logistische Regression

Hinweise

Ausgabe erstellt		11-MAR-2026 18:19:50
Kommentare		
Eingabe	Daten	H:\Gabapentin Auswertung\28.02.24\6.1.2022 Gabapentin_1.sav
	Aktiver Datensatz	DataSet1
	Filter	<keine>
	Gewichtung	<keine>
	Aufgeteilte Datei	<keine>
	Anzahl der Zeilen in der Arbeitsdatei	93
Behandlung fehlender Werte	Definition für "fehlend"	Benutzerdefinierte fehlende Werte werden als fehlend behandelt
Syntax		LOGISTIC REGRESSION VARIABLES SUD1_0 /METHOD=ENTER Alter_korr Schulabschluss_korr Geschlecht /CONTRAST (Geschlecht)=Indicator(1) /CONTRAST (Schulabschluss_korr)=Indicator(4) /CONTRAST (Alter_korr)=Indicator(1) /PRINT=CI(95) /CRITERIA=PIN(0.05) POUT(0.10) ITERATE(20) CUT(0.5).

Ressourcen	Prozessorzeit	00:00:00,02
	Verstrichene Zeit	00:00:00,01

Zusammenfassung der Fallverarbeitung

Ungewichtete Fälle[a]		N	Prozent
Ausgewählte Fälle	Einbezogen in Analyse	93	100,0
	Fehlende Fälle	0	,0
	Gesamt	93	100,0
Nicht ausgewählte Fälle		0	,0
Gesamt		93	100,0

a. Wenn die Gewichtung wirksam ist, finden Sie die Gesamtzahl der Fälle in der Klassifizierungstabelle.

Codierung abhängiger Variablen

Ursprünglicher Wert	Interner Wert
0	0
1	1

Codierungen kategorialer Variablen

			Parametercodierung		
		Häufigkeit	(1)	(2)	(3)
Schulabschluss_korr	1	5	1,000	,000	,000
	2	49	,000	1,000	,000
	3	16	,000	,000	1,000
	4	23	,000	,000	,000
Alter_korr	1	40	,000	,000	
	2	28	1,000	,000	
	3	25	,000	1,000	
1=m, 0=w	weiblich	48	,000		
	männlich	45	1,000		

Block 0: Anfangsblock

Klassifizierungstabelle[a,b]

			Vorhergesagt		
			SUD1_0		Prozentsatz der Richtigen
Beobachtet			0	1	
Schritt 0	SUD1_0	0	64	0	100,0
		1	29	0	,0
	Gesamtprozentsatz				68,8

a. Konstante in das Modell einbezogen.

b. Der Trennwert lautet ,500

Variablen in der Gleichung

		RegressionskoeffizientB	Standardfehler	Wald	df	Sig.	Exp(B)
Schritt 0	Konstante	-,792	,224	12,505	1	<,001	,453

Variablen nicht in der Gleichung

			Wert	df	Sig.
Schritt 0	Variablen	Alter_korr	,801	2	,670
		Alter_korr(1)	,714	1	,398
		Alter_korr(2)	,370	1	,543
		Schulabschluss_korr	6,440	3	,092
		Schulabschluss_korr(1)	2,045	1	,153
		Schulabschluss_korr(2)	5,603	1	,018
		Schulabschluss_korr(3)	1,422	1	,233
		1=m, 0=w(1)	4,951	1	,026
	Gesamtstatistik		13,446	6	,036

Block 1: Methode = Einschluß

Omnibus-Tests der Modellkoeffizienten

		Chi-Quadrat	df	Sig.
Schritt 1	Schritt	14,185	6	,028
	Block	14,185	6	,028
	Modell	14,185	6	,028

Modellzusammenfassung

Schritt	-2 Log-Likelihood	Cox & Snell R-Quadrat	Nagelkerkes R-Quadrat
1	101,238[a]	,141	,199

a. Schätzung beendet bei Iteration Nummer 4, weil die Parameterschätzer sich um weniger als ,001 änderten.

Klassifizierungstabelle[a]

			Vorhergesagt		
			SUD1_0		Prozentsatz der Richtigen
	Beobachtet		0	1	
Schritt 1	SUD1_0	0	54	10	84,4
		1	16	13	44,8
	Gesamtprozentsatz				72,0

a. Der Trennwert lautet ,500

Variablen in der Gleichung

		RegressionskoeffizientB	Standardfehler	Wald	df
Schritt 1[a]	Alter_korr			,043	2
	Alter_korr(1)	-,045	,610	,006	1
	Alter_korr(2)	,089	,597	,022	1

	Schulabschluss_korr			7,748	3
	Schulabschluss_korr(1)	1,396	1,089	1,644	1
	Schulabschluss_korr(2)	-,995	,594	2,802	1
	Schulabschluss_korr(3)	,442	,714	,384	1
	1=m, 0=w(1)	1,380	,532	6,738	1
	Konstante	-1,241	,627	3,920	1

Variablen in der Gleichung

				95% Konfidenzintervall für EXP(B)	
		Sig.	Exp(B)	Unterer Wert	Oberer Wert
Schritt 1[a]	Alter_korr	,979			
	Alter_korr(1)	,941	,956	,289	3,159
	Alter_korr(2)	,881	1,094	,340	3,522
	Schulabschluss_korr	,052			
	Schulabschluss_korr(1)	,200	4,041	,478	34,166
	Schulabschluss_korr(2)	,094	,370	,115	1,185
	Schulabschluss_korr(3)	,536	1,556	,384	6,306
	1=m, 0=w(1)	,009	3,974	1,402	11,264
	Konstante	,048	,289		

a. In Schritt 1 eingegebene Variablen: Alter_korr, Schulabschluss_korr, 1=m, 0=w.

Logistische Regression

Hinweise

Ausgabe erstellt		11-MAR-2026 18:19:50
Kommentare		
Eingabe	Daten	H:\Gabapentin Auswertung\28.02.24\6.1.2022 Gabapentin_1.sav
	Aktiver Datensatz	DataSet1
	Filter	<keine>
	Gewichtung	<keine>
	Aufgeteilte Datei	<keine>
	Anzahl der Zeilen in der Arbeitsdatei	93
Behandlung fehlender Werte	Definition für "fehlend"	Benutzerdefinierte fehlende Werte werden als fehlend behandelt
Syntax		LOGISTIC REGRESSION VARIABLES SUD1_0 /METHOD=ENTER Schmerzdauer_korr NeuropSchmerz_kor SchmerzKopf_kor SchmerzRücken_kor SchmerzNacken_kor SchmerzArm_kor SchmerzGelenk_kor SchmerzGesicht_kor SchmerzBein_kor Benzo_kor Opioide_kor /CONTRAST (Schmerzdauer_korr)=Indicator(1) /PRINT=CI(95) /CRITERIA=PIN(0.05) POUT(0.10) ITERATE(20) CUT(0.5).
Ressourcen	Prozessorzeit	00:00:00,02
	Verstrichene Zeit	00:00:00,01

Zusammenfassung der Fallverarbeitung

Ungewichtete Fälle[a]		N	Prozent
Ausgewählte Fälle	Einbezogen in Analyse	93	100,0
	Fehlende Fälle	0	,0
	Gesamt	93	100,0
Nicht ausgewählte Fälle		0	,0
Gesamt		93	100,0

a. Wenn die Gewichtung wirksam ist, finden Sie die Gesamtzahl der Fälle in der Klassifizierungstabelle.

Codierung abhängiger Variablen

Ursprünglicher Wert	Interner Wert
0	0
1	1

Codierungen kategorialer Variablen

		Häufigkeit	Parametercodierung (1)	(2)	(3)	(4)
Schmerzdauer_korr	1	11	,000	,000	,000	,000
	2	4	1,000	,000	,000	,000
	3	1	,000	1,000	,000	,000
	4	36	,000	,000	1,000	,000
	5	41	,000	,000	,000	1,000

```
Block 0: Anfangsblock
```

Klassifizierungstabelle[a,b]

Beobachtet			Vorhergesagt SUD1_0: 0	1	Prozentsatz der Richtigen
Schritt 0	SUD1_0	0	64	0	100,0
		1	29	0	,0

	Gesamtprozentsatz			68,8

a. Konstante in das Modell einbezogen.

b. Der Trennwert lautet ,500

Variablen in der Gleichung

		Regressionskoeffizient B	Standardfehler	Wald	df	Sig.	Exp(B)
Schritt 0	Konstante	-,792	,224	12,505	1	<,001	,453

Variablen nicht in der Gleichung

			Wert	df	Sig.
Schritt 0	Variablen	Schmerzdauer_korr	2,732	4	,604
		Schmerzdauer_korr(1)	,690	1	,406
		Schmerzdauer_korr(2)	,458	1	,499
		Schmerzdauer_korr(3)	,317	1	,573
		Schmerzdauer_korr(4)	,997	1	,318
		NeuropSchmerz_kor	1,392	1	,238
		SchmerzKopf_kor	,048	1	,826
		SchmerzRücken_kor	,709	1	,400
		SchmerzNacken_kor	4,855	1	,028
		SchmerzArm_kor	,447	1	,504
		SchmerzGelenk_kor	2,162	1	,141
		SchmerzGesicht_kor	1,007	1	,316
		SchmerzBein_kor	,975	1	,324
		Benzo_kor	,021	1	,883
		Opioide_kor	,555	1	,456
	Gesamtstatistik		15,917	14	,318

Block 1: Methode = Einschluß

Omnibus-Tests der Modellkoeffizienten

		Chi-Quadrat	df	Sig.
Schritt 1	Schritt	17,375	14	,237
	Block	17,375	14	,237
	Modell	17,375	14	,237

Modellzusammenfassung

Schritt	-2 Log-Likelihood	Cox & Snell R-Quadrat	Nagelkerkes R-Quadrat
1	98,048[a]	,170	,240

a. Schätzung beendet bei Iteration Nummer 20 weil die Höchstzahl der Iterationen erreicht wurde. Endlösung kann nicht gefunden werden.

Klassifizierungstabelle[a]

			Vorhergesagt		
			SUD1_0		Prozentsatz der Richtigen
	Beobachtet		0	1	
Schritt 1	SUD1_0	0	57	7	89,1
		1	19	10	34,5
	Gesamtprozentsatz				72,0

a. Der Trennwert lautet ,500

Variablen in der Gleichung

		RegressionskoeffizientB	Standardfehler	Wald	df
Schritt 1[a]	Schmerzdauer_korr			2,960	4
	Schmerzdauer_korr(1)	2,724	1,626	2,807	1
	Schmerzdauer_korr(2)	-18,901	40192,970	,000	1
	Schmerzdauer_korr(3)	,716	1,026	,487	1
	Schmerzdauer_korr(4)	,968	1,000	,938	1

	NeuropSchmerz_kor	1,363	,849	2,578	1
	SchmerzKopf_kor	,795	,885	,806	1
	SchmerzRücken_kor	,458	,587	,611	1
	SchmerzNacken_kor	,918	,684	1,801	1
	SchmerzArm_kor	,037	,618	,004	1
	SchmerzGelenk_kor	,491	,693	,502	1
	SchmerzGesicht_kor	-2,242	1,503	2,226	1
	SchmerzBein_kor	,517	,709	,531	1
	Benzo_kor	,751	,986	,581	1
	Opioide_kor	-1,056	,610	2,992	1
	Konstante	-3,780	1,435	6,936	1

Variablen in der Gleichung

				95% Konfidenzintervall für EXP(B)	
		Sig.	Exp(B)	Unterer Wert	Oberer Wert
Schritt 1[a]	Schmerzdauer_korr	,565			
	Schmerzdauer_korr(1)	,094	15,238	,630	368,755
	Schmerzdauer_korr(2)	1,000	,000	,000	.
	Schmerzdauer_korr(3)	,485	2,046	,274	15,289
	Schmerzdauer_korr(4)	,333	2,633	,371	18,678
	NeuropSchmerz_kor	,108	3,906	,740	20,611
	SchmerzKopf_kor	,369	2,214	,391	12,554
	SchmerzRücken_kor	,435	1,582	,501	4,995
	SchmerzNacken_kor	,180	2,503	,655	9,562
	SchmerzArm_kor	,952	1,038	,309	3,486
	SchmerzGelenk_kor	,479	1,634	,420	6,357
	SchmerzGesicht_kor	,136	,106	,006	2,020
	SchmerzBein_kor	,466	1,676	,417	6,731
	Benzo_kor	,446	2,120	,307	14,631
	Opioide_kor	,084	,348	,105	1,151
	Konstante	,008	,023		

a. In Schritt 1 eingegebene Variablen: Schmerzdauer_korr, NeuropSchmerz_kor, SchmerzKopf_kor, SchmerzRücken_kor, SchmerzNacken_kor, SchmerzArm_kor, SchmerzGelenk_kor, SchmerzGesicht_kor, SchmerzBein_kor, Benzo_kor, Opioide_kor.

Logistische Regression

Hinweise

Ausgabe erstellt		11-MAR-2026 18:19:50
Kommentare		
Eingabe	Daten	H:\Gabapentin Auswertung\28.02.24\6.1.2022 Gabapentin_1.sav
	Aktiver Datensatz	DataSet1
	Filter	<keine>
	Gewichtung	<keine>
	Aufgeteilte Datei	<keine>
	Anzahl der Zeilen in der Arbeitsdatei	93
Behandlung fehlender Werte	Definition für "fehlend"	Benutzerdefinierte fehlende Werte werden als fehlend behandelt
Syntax		LOGISTIC REGRESSION VARIABLES SUD1_0 /METHOD=ENTER DSM_1_kor DSM_2_kor DSM_3_kor DSM_4_kor DSM_5_kor DSM_6_kor DSM_7_kor DSM_8_kor DSM_9_kor DSM_10_kor DSM_11_kor /CONTRAST (DSM_1_kor)=Indicator(1) /CONTRAST

		(DSM_2_kor)=Indicator(1)<br>/CONTRAST (DSM_3_kor)=Indicator(1)<br>/CONTRAST (DSM_4_kor)=Indicator(1)<br>/CONTRAST (DSM_5_kor)=Indicator(1)<br>/CONTRAST (DSM_6_kor)=Indicator(1)<br>/CONTRAST (DSM_7_kor)=Indicator(1)<br>/CONTRAST (DSM_8_kor)=Indicator(1)<br>/CONTRAST (DSM_9_kor)=Indicator(1)<br>/CONTRAST (DSM_10_kor)=Indicator(1)<br>/CONTRAST (DSM_11_kor)=Indicator(1)<br>/PRINT=CI(95)<br>/CRITERIA=PIN(0.05) POUT(0.10) ITERATE(20) CUT(0.5).
Ressourcen	Prozessorzeit	00:00:00,02
	Verstrichene Zeit	00:00:00,01

Zusammenfassung der Fallverarbeitung

Ungewichtete Fälle[a]		N	Prozent
Ausgewählte Fälle	Einbezogen in Analyse	93	100,0
	Fehlende Fälle	0	,0

Gesamt	93	100,0
Nicht ausgewählte Fälle	0	,0
Gesamt	93	100,0

a. Wenn die Gewichtung wirksam ist, finden Sie die Gesamtzahl der Fälle in der Klassifizierungstabelle.

Codierung abhängiger Variablen

Ursprünglicher Wert	Interner Wert
0	0
1	1

Codierungen kategorialer Variablen

		Häufigkeit	Parametercodierung (1)
DSM_11_kor	0	77	,000
	1	16	1,000
DSM_2_kor	0	60	,000
	1	33	1,000
DSM_3_kor	0	84	,000
	1	9	1,000
DSM_4_kor	0	88	,000
	1	5	1,000
DSM_5_kor	0	89	,000
	1	4	1,000
DSM_6_kor	0	92	,000
	1	1	1,000
DSM_7_kor	0	81	,000
	1	12	1,000
DSM_10_kor	0	83	,000
	1	10	1,000
DSM_9_kor	0	78	,000
	1	15	1,000
DSM_8_kor	0	87	,000
	1	6	1,000
DSM_1_kor	0	65	,000

1	28	1,000

Block 0: Anfangsblock

Klassifizierungstabelle[a,b]

		Beobachtet	Vorhergesagt SUD1_0 0	Vorhergesagt SUD1_0 1	Prozentsatz der Richtigen
Schritt 0	SUD1_0	0	64	0	100,0
		1	29	0	,0
	Gesamtprozentsatz				68,8

a. Konstante in das Modell einbezogen.

b. Der Trennwert lautet ,500

Variablen in der Gleichung

		RegressionskoeffizientB	Standardfehler	Wald	df	Sig.	Exp(B)
Schritt 0	Konstante	-,792	,224	12,505	1	<,001	,453

Variablen nicht in der Gleichung

			Wert	df	Sig.
Schritt 0	Variablen	DSM_1_kor(1)	12,581	1	<,001
		DSM_2_kor(1)	35,357	1	<,001
		DSM_3_kor(1)	15,462	1	<,001
		DSM_4_kor(1)	11,661	1	<,001
		DSM_5_kor(1)	9,224	1	,002
		DSM_6_kor(1)	2,231	1	,135
		DSM_7_kor(1)	30,406	1	<,001
		DSM_8_kor(1)	8,129	1	,004
		DSM_9_kor(1)	32,193	1	<,001
		DSM_10_kor(1)	1,849	1	,174
		DSM_11_kor(1)	5,659	1	,017

	Gesamtstatistik	71,163	11	<,001

Block 1: Methode = Einschluß

Omnibus-Tests der Modellkoeffizienten

		Chi-Quadrat	df	Sig.
Schritt 1	Schritt	115,423	11	<,001
	Block	115,423	11	<,001
	Modell	115,423	11	<,001

Modellzusammenfassung

Schritt	-2 Log-Likelihood	Cox & Snell R-Quadrat	Nagelkerkes R-Quadrat
1	,000[a]	,711	1,000

a. Schätzung beendet bei Iteration Nummer 20 weil die Höchstzahl der Iterationen erreicht wurde. Endlösung kann nicht gefunden werden.

Klassifizierungstabelle[a]

Beobachtet			Vorhergesagt SUD1_0 0	1	Prozentsatz der Richtigen
Schritt 1	SUD1_0	0	64	0	100,0
		1	0	29	100,0
	Gesamtprozentsatz				100,0

a. Der Trennwert lautet ,500

Variablen in der Gleichung

		RegressionskoeffizientB	Standardfehler	Wald	df	Sig.
Schritt 1[a]	DSM_1_kor(1)	37,297	5840,058	,000	1	,995
	DSM_2_kor(1)	37,745	5559,834	,000	1	,995

	DSM_3_kor(1)	36,933	11050,488	,000	1	,997
	DSM_4_kor(1)	,903	14882,027	,000	1	1,000
	DSM_5_kor(1)	-,474	20884,381	,000	1	1,000
	DSM_6_kor(1)	-142,380	116558,802	,000	1	,999
	DSM_7_kor(1)	38,876	14691,489	,000	1	,998
	DSM_8_kor(1)	37,447	10528,347	,000	1	,997
	DSM_9_kor(1)	37,911	8267,411	,000	1	,996
	DSM_10_kor(1)	5,811	105643,011	,000	1	1,000
	DSM_11_kor(1)	-6,073	106013,422	,000	1	1,000
	Konstante	-56,554	6819,583	,000	1	,993

Variablen in der Gleichung

			95% Konfidenzintervall für EXP(B)	
		Exp(B)	Unterer Wert	Oberer Wert
Schritt 1[a]	DSM_1_kor(1)	15771215121592090,000	,000	.
	DSM_2_kor(1)	24678830190885872,000	,000	.
	DSM_3_kor(1)	10959807178680608,000	,000	.
	DSM_4_kor(1)	2,468	,000	.
	DSM_5_kor(1)	,623	,000	.
	DSM_6_kor(1)	,000	,000	.
	DSM_7_kor(1)	76482120447313792,000	,000	.
	DSM_8_kor(1)	18326308828902516,000	,000	.
	DSM_9_kor(1)	29152111114315384,000	,000	.
	DSM_10_kor(1)	333,825	,000	.
	DSM_11_kor(1)	,002	,000	.
	Konstante	,000		

a. In Schritt 1 eingegebene Variablen: DSM_1_kor, DSM_2_kor, DSM_3_kor, DSM_4_kor, DSM_5_kor, DSM_6_kor, DSM_7_kor, DSM_8_kor, DSM_9_kor, DSM_10_kor, DSM_11_kor.

Logistische Regression

Hinweise

Ausgabe erstellt		11-MAR-2026 18:19:50
Kommentare		
Eingabe	Daten	H:\Gabapentin Auswertung\28.02.24\6.1.2022 Gabapentin_1.sav
	Aktiver Datensatz	DataSet1
	Filter	<keine>
	Gewichtung	<keine>
	Aufgeteilte Datei	<keine>
	Anzahl der Zeilen in der Arbeitsdatei	93
Behandlung fehlender Werte	Definition für "fehlend"	Benutzerdefinierte fehlende Werte werden als fehlend behandelt
Syntax		LOGISTIC REGRESSION VARIABLES SUD1_0 /METHOD=ENTER DSM_1_kor /CONTRAST (DSM_1_kor)=Indicator(1) /PRINT=CI(95) /CRITERIA=PIN(0.05) POUT(0.10) ITERATE(20) CUT(0.5).
Ressourcen	Prozessorzeit	00:00:00,00
	Verstrichene Zeit	00:00:00,00

Zusammenfassung der Fallverarbeitung

Ungewichtete Fälle[a]		N	Prozent
Ausgewählte Fälle	Einbezogen in Analyse	93	100,0
	Fehlende Fälle	0	,0
	Gesamt	93	100,0
Nicht ausgewählte Fälle		0	,0
Gesamt		93	100,0

a. Wenn die Gewichtung wirksam ist, finden Sie die Gesamtzahl der Fälle in der Klassifizierungstabelle.

Codierung abhängiger Variablen

Ursprünglicher Wert	Interner Wert
0	0
1	1

Codierungen kategorialer Variablen

		Häufigkeit	Parametercodierung (1)
DSM_1_kor	0	65	,000
	1	28	1,000

```
Block 0: Anfangsblock
```

Klassifizierungstabelle[a,b]

Beobachtet			Vorhergesagt SUD1_0: 0	Vorhergesagt SUD1_0: 1	Prozentsatz der Richtigen
Schritt 0	SUD1_0	0	64	0	100,0
		1	29	0	,0
	Gesamtprozentsatz				68,8

a. Konstante in das Modell einbezogen.

b. Der Trennwert lautet ,500

Variablen in der Gleichung

		Regressionskoeffizient B	Standardfehler	Wald	df	Sig.	Exp(B)
Schritt 0	Konstante	-,792	,224	12,505	1	<,001	,453

Variablen nicht in der Gleichung

			Wert	df	Sig.
Schritt 0	Variablen	DSM_1_kor(1)	12,581	1	<,001
	Gesamtstatistik		12,581	1	<,001

```
Block 1: Methode = Einschluß
```

Omnibus-Tests der Modellkoeffizienten

		Chi-Quadrat	df	Sig.
Schritt 1	Schritt	12,128	1	<,001
	Block	12,128	1	<,001
	Modell	12,128	1	<,001

Modellzusammenfassung

Schritt	-2 Log-Likelihood	Cox & Snell R-Quadrat	Nagelkerkes R-Quadrat
1	103,295[a]	,122	,172

a. Schätzung beendet bei Iteration Nummer 4, weil die Parameterschätzer sich um weniger als ,001 änderten.

Klassifizierungstabelle[a]

Beobachtet	Vorhergesagt

			SUD1_0		Prozentsatz der Richtigen
			0	1	
Schritt 1	SUD1_0	0	52	12	81,3
		1	13	16	55,2
	Gesamtprozentsatz				73,1

a. Der Trennwert lautet ,500

Variablen in der Gleichung

		Regressionskoeffizient B	Standardfehler	Wald	df	Sig.
Schritt 1[a]	DSM_1_kor(1)	1,674	,492	11,580	1	<,001
	Konstante	-1,386	,310	19,987	1	<,001

Variablen in der Gleichung

		Exp(B)	95% Konfidenzintervall für EXP(B)	
			Unterer Wert	Oberer Wert
Schritt 1[a]	DSM_1_kor(1)	5,333	2,034	13,987
	Konstante	,250		

a. In Schritt 1 eingegebene Variablen: DSM_1_kor.

Logistische Regression

Hinweise

Ausgabe erstellt		11-MAR-2026 18:19:50
Kommentare		
Eingabe	Daten	H:\Gabapentin Auswertung\28.02.24\6.

		1.2022<br>Gabapentin_1.sav
	Aktiver Datensatz	DataSet1
	Filter	<keine>
	Gewichtung	<keine>
	Aufgeteilte Datei	<keine>
	Anzahl der Zeilen in der Arbeitsdatei	93
Behandlung fehlender Werte	Definition für "fehlend"	Benutzerdefinierte fehlende Werte werden als fehlend behandelt
Syntax		LOGISTIC REGRESSION VARIABLES SUD1_0 /METHOD=ENTER DSM_2_kor /CONTRAST (DSM_2_kor)=Indicator(1) /PRINT=CI(95) /CRITERIA=PIN(0.05) POUT(0.10) ITERATE(20) CUT(0.5).
Ressourcen	Prozessorzeit	00:00:00,00
	Verstrichene Zeit	00:00:00,01

Zusammenfassung der Fallverarbeitung

Ungewichtete Fälle[a]		N	Prozent
Ausgewählte Fälle	Einbezogen in Analyse	93	100,0
	Fehlende Fälle	0	,0
	Gesamt	93	100,0
Nicht ausgewählte Fälle		0	,0
Gesamt		93	100,0

a. Wenn die Gewichtung wirksam ist, finden Sie die Gesamtzahl der Fälle in der Klassifizierungstabelle.

Codierung abhängiger

Variablen

Ursprünglicher Wert	Interner Wert
0	0
1	1

Codierungen kategorialer Variablen

		Häufigkeit	Parametercodierung (1)
DSM_2_kor	0	60	,000
	1	33	1,000

```
Block 0: Anfangsblock
```

Klassifizierungstabelle[a,b]

	Beobachtet		Vorhergesagt SUD1_0 0	Vorhergesagt SUD1_0 1	Prozentsatz der Richtigen
Schritt 0	SUD1_0	0	64	0	100,0
		1	29	0	,0
	Gesamtprozentsatz				68,8

a. Konstante in das Modell einbezogen.

b. Der Trennwert lautet ,500

Variablen in der Gleichung

		RegressionskoeffizientB	Standardfehler	Wald	df	Sig.	Exp(B)
Schritt 0	Konstante	-,792	,224	12,505	1	<,001	,453

Variablen nicht in der Gleichung

			Wert	df	Sig.
Schritt 0	Variablen	DSM_2_kor(1	35,357	1	<,001

	)			
	Gesamtstatistik	35,357	1	<,001

Block 1: Methode = Einschluß

Omnibus-Tests der Modellkoeffizienten

		Chi-Quadrat	df	Sig.
Schritt 1	Schritt	35,928	1	<,001
	Block	35,928	1	<,001
	Modell	35,928	1	<,001

Modellzusammenfassung

Schritt	-2 Log-Likelihood	Cox & Snell R-Quadrat	Nagelkerkes R-Quadrat
1	79,495[a]	,320	,451

a. Schätzung beendet bei Iteration Nummer 5, weil die Parameterschätzer sich um weniger als ,001 änderten.

Klassifizierungstabelle[a]

	Beobachtet		Vorhergesagt SUD1_0 0	Vorhergesagt SUD1_0 1	Prozentsatz der Richtigen
Schritt 1	SUD1_0	0	54	10	84,4
		1	6	23	79,3
	Gesamtprozentsatz				82,8

a. Der Trennwert lautet ,500

Variablen in der Gleichung

		RegressionskoeffizientB	Standardfehler	Wald	df	Sig.
Schritt 1[a]	DSM_2_kor(1	3,030	,573	27,937	1	<,001

	)					
	Konstante	-2,197	,430	26,070	1	<,001

Variablen in der Gleichung

			95% Konfidenzintervall für EXP(B)	
		Exp(B)	Unterer Wert	Oberer Wert
Schritt 1[a]	DSM_2_kor(1)	20,700	6,730	63,673
	Konstante	,111		

a. In Schritt 1 eingegebene Variablen: DSM_2_kor.

```
Logistische Regression
```

Hinweise

Ausgabe erstellt		11-MAR-2026 18:19:50
Kommentare		
Eingabe	Daten	H:\Gabapentin Auswertung\28.02.24\6.1.2022 Gabapentin_1.sav
	Aktiver Datensatz	DataSet1
	Filter	<keine>
	Gewichtung	<keine>
	Aufgeteilte Datei	<keine>
	Anzahl der Zeilen in der Arbeitsdatei	93
Behandlung fehlender Werte	Definition für "fehlend"	Benutzerdefinierte fehlende Werte werden als fehlend behandelt
Syntax		LOGISTIC REGRESSION VARIABLES SUD1_0

		/METHOD=ENTER DSM_3_kor /CONTRAST (DSM_3_kor)=Indicator(1) /PRINT=CI(95) /CRITERIA=PIN(0.05) POUT(0.10) ITERATE(20) CUT(0.5).
Ressourcen	Prozessorzeit	00:00:00,00
	Verstrichene Zeit	00:00:00,01

Zusammenfassung der Fallverarbeitung

Ungewichtete Fälle[a]		N	Prozent
Ausgewählte Fälle	Einbezogen in Analyse	93	100,0
	Fehlende Fälle	0	,0
	Gesamt	93	100,0
Nicht ausgewählte Fälle		0	,0
Gesamt		93	100,0

a. Wenn die Gewichtung wirksam ist, finden Sie die Gesamtzahl der Fälle in der Klassifizierungstabelle.

Codierung abhängiger Variablen

Ursprünglicher Wert	Interner Wert
0	0
1	1

Codierungen kategorialer Variablen

		Häufigkeit	Parametercodierung (1)
DSM_3_kor	0	84	,000
	1	9	1,000

Block 0: Anfangsblock

Klassifizierungstabelle[a,b]

Beobachtet			Vorhergesagt SUD1_0 0	1	Prozentsatz der Richtigen
Schritt 0	SUD1_0	0	64	0	100,0
		1	29	0	,0
	Gesamtprozentsatz				68,8

a. Konstante in das Modell einbezogen.

b. Der Trennwert lautet ,500

Variablen in der Gleichung

		RegressionskoeffizientB	Standardfehler	Wald	df	Sig.	Exp(B)
Schritt 0	Konstante	-,792	,224	12,505	1	<,001	,453

Variablen nicht in der Gleichung

			Wert	df	Sig.
Schritt 0	Variablen	DSM_3_kor(1)	15,462	1	<,001
	Gesamtstatistik		15,462	1	<,001

Block 1: Methode = Einschluß

Omnibus-Tests der Modellkoeffizienten

		Chi-Quadrat	df	Sig.
Schritt 1	Schritt	14,672	1	<,001
	Block	14,672	1	<,001
	Modell	14,672	1	<,001

Modellzusammenfassung

Schritt	-2 Log-Likelihood	Cox & Snell R-Quadrat	Nagelkerkes R-Quadrat
1	100,751[a]	,146	,205

a. Schätzung beendet bei Iteration Nummer 5, weil die Parameterschätzer sich um weniger als ,001 änderten.

Klassifizierungstabelle[a]

Beobachtet			Vorhergesagt SUD1_0: 0	Vorhergesagt SUD1_0: 1	Prozentsatz der Richtigen
Schritt 1	SUD1_0	0	63	1	98,4
		1	21	8	27,6
	Gesamtprozentsatz				76,3

a. Der Trennwert lautet ,500

Variablen in der Gleichung

		RegressionskoeffizientB	Standardfehler	Wald	df	Sig.
Schritt 1[a]	DSM_3_kor(1)	3,178	1,090	8,498	1	,004
	Konstante	-1,099	,252	19,009	1	<,001

Variablen in der Gleichung

		Exp(B)	95% Konfidenzintervall für EXP(B): Unterer Wert	95% Konfidenzintervall für EXP(B): Oberer Wert
Schritt 1[a]	DSM_3_kor(1)	24,000	2,833	203,317
	Konstante	,333		

a. In Schritt 1 eingegebene Variablen: DSM_3_kor.

Logistische Regression

Hinweise

Ausgabe erstellt		11-MAR-2026 18:19:50
Kommentare		
Eingabe	Daten	H:\Gabapentin Auswertung\28.02.24\6.1.2022 Gabapentin_1.sav
	Aktiver Datensatz	DataSet1
	Filter	<keine>
	Gewichtung	<keine>
	Aufgeteilte Datei	<keine>
	Anzahl der Zeilen in der Arbeitsdatei	93
Behandlung fehlender Werte	Definition für "fehlend"	Benutzerdefinierte fehlende Werte werden als fehlend behandelt
Syntax		LOGISTIC REGRESSION VARIABLES SUD1_0 /METHOD=ENTER DSM_4_kor /CONTRAST (DSM_4_kor)=Indicator(1) /PRINT=CI(95) /CRITERIA=PIN(0.05) POUT(0.10) ITERATE(20) CUT(0.5).
Ressourcen	Prozessorzeit	00:00:00,02
	Verstrichene Zeit	00:00:00,00

Zusammenfassung der Fallverarbeitung

Ungewichtete Fälle[a]		N	Prozent
Ausgewählte Fälle	Einbezogen in Analyse	93	100,0
	Fehlende Fälle	0	,0
	Gesamt	93	100,0
Nicht ausgewählte Fälle		0	,0
Gesamt		93	100,0

a. Wenn die Gewichtung wirksam ist, finden Sie die Gesamtzahl der Fälle in der Klassifizierungstabelle.

Codierung abhängiger Variablen

Ursprünglicher Wert	Interner Wert
0	0
1	1

Codierungen kategorialer Variablen

		Häufigkeit	Parametercodierung (1)
DSM_4_kor	0	88	,000
	1	5	1,000

```
Block 0: Anfangsblock
```

Klassifizierungstabelle[a,b]

Beobachtet			Vorhergesagt SUD1_0: 0	Vorhergesagt SUD1_0: 1	Prozentsatz der Richtigen
Schritt 0	SUD1_0	0	64	0	100,0
		1	29	0	,0
	Gesamtprozentsatz				68,8

a. Konstante in das Modell einbezogen.

b. Der Trennwert lautet ,500

Variablen in der Gleichung

		RegressionskoeffizientB	Standardfehler	Wald	df	Sig.	Exp(B)
Schritt 0	Konstante	-,792	,224	12,505	1	<,001	,453

Variablen nicht in der Gleichung

			Wert	df	Sig.
Schritt 0	Variablen	DSM_4_kor(1)	11,661	1	<,001
	Gesamtstatistik		11,661	1	<,001

```
Block 1: Methode = Einschluß
```

Omnibus-Tests der Modellkoeffizienten

		Chi-Quadrat	df	Sig.
Schritt 1	Schritt	12,296	1	<,001
	Block	12,296	1	<,001
	Modell	12,296	1	<,001

Modellzusammenfassung

Schritt	-2 Log-Likelihood	Cox & Snell R-Quadrat	Nagelkerkes R-Quadrat
1	103,128[a]	,124	,174

a. Schätzung beendet bei Iteration Nummer 20 weil die Höchstzahl der Iterationen erreicht wurde. Endlösung kann nicht gefunden werden.

Klassifizierungstabelle[a]

		Vorhergesagt		
		SUD1_0		Prozentsatz der Richtigen
Beobachtet		0	1	

Schritt 1	SUD1_0	0	64	0	100,0
		1	24	5	17,2
	Gesamtprozentsatz				74,2

a. Der Trennwert lautet ,500

Variablen in der Gleichung

		RegressionskoeffizientB	Standardfehler	Wald	df	Sig.
Schritt 1[a]	DSM_4_kor(1)	22,184	17974,842	,000	1	,999
	Konstante	-,981	,239	16,792	1	<,001

Variablen in der Gleichung

			95% Konfidenzintervall für EXP(B)	
		Exp(B)	Unterer Wert	Oberer Wert
Schritt 1[a]	DSM_4_kor(1)	4307932914,270	,000	.
	Konstante	,375		

a. In Schritt 1 eingegebene Variablen: DSM_4_kor.

```
Logistische Regression
```

Hinweise

Ausgabe erstellt		11-MAR-2026 18:19:50
Kommentare		
Eingabe	Daten	H:\Gabapentin Auswertung\28.02.24\6.1.2022 Gabapentin_1.sav

	Aktiver Datensatz	DataSet1
	Filter	<keine>
	Gewichtung	<keine>
	Aufgeteilte Datei	<keine>
	Anzahl der Zeilen in der Arbeitsdatei	93
Behandlung fehlender Werte	Definition für "fehlend"	Benutzerdefinierte fehlende Werte werden als fehlend behandelt
Syntax		LOGISTIC REGRESSION VARIABLES SUD1_0 /METHOD=ENTER DSM_5_kor /CONTRAST (DSM_5_kor)=Indicator(1) /PRINT=CI(95) /CRITERIA=PIN(0.05) POUT(0.10) ITERATE(20) CUT(0.5).
Ressourcen	Prozessorzeit	00:00:00,00
	Verstrichene Zeit	00:00:00,01

Zusammenfassung der Fallverarbeitung

Ungewichtete Fälle[a]		N	Prozent
Ausgewählte Fälle	Einbezogen in Analyse	93	100,0
	Fehlende Fälle	0	,0
	Gesamt	93	100,0
Nicht ausgewählte Fälle		0	,0
Gesamt		93	100,0

a. Wenn die Gewichtung wirksam ist, finden Sie die Gesamtzahl der Fälle in der Klassifizierungstabelle.

Codierung abhängiger Variablen

Ursprünglicher	Interner

Wert	Wert
0	0
1	1

Codierungen kategorialer Variablen

		Häufigkeit	Parametercodierung (1)
DSM_5_kor	0	89	,000
	1	4	1,000

```
Block 0: Anfangsblock
```

Klassifizierungstabelle[a,b]

	Beobachtet		Vorhergesagt SUD1_0 0	Vorhergesagt SUD1_0 1	Prozentsatz der Richtigen
Schritt 0	SUD1_0	0	64	0	100,0
		1	29	0	,0
	Gesamtprozentsatz				68,8

a. Konstante in das Modell einbezogen.

b. Der Trennwert lautet ,500

Variablen in der Gleichung

		RegressionskoeffizientB	Standardfehler	Wald	df	Sig.	Exp(B)
Schritt 0	Konstante	-,792	,224	12,505	1	<,001	,453

Variablen nicht in der Gleichung

			Wert	df	Sig.
Schritt 0	Variablen	DSM_5_kor(1)	9,224	1	,002
	Gesamtstatistik		9,224	1	,002

Block 1: Methode = Einschluß

Omnibus-Tests der Modellkoeffizienten

		Chi-Quadrat	df	Sig.
Schritt 1	Schritt	9,727	1	,002
	Block	9,727	1	,002
	Modell	9,727	1	,002

Modellzusammenfassung

Schritt	-2 Log-Likelihood	Cox & Snell R-Quadrat	Nagelkerkes R-Quadrat
1	105,696[a]	,099	,140

a. Schätzung beendet bei Iteration Nummer 20 weil die Höchstzahl der Iterationen erreicht wurde. Endlösung kann nicht gefunden werden.

Klassifizierungstabelle[a]

	Beobachtet		Vorhergesagt SUD1_0 0	Vorhergesagt SUD1_0 1	Prozentsatz der Richtigen
Schritt 1	SUD1_0	0	64	0	100,0
		1	25	4	13,8
	Gesamtprozentsatz				73,1

a. Der Trennwert lautet ,500

Variablen in der Gleichung

		RegressionskoeffizientB	Standardfehler	Wald	df	Sig.
Schritt 1[a]	DSM_5_kor(1)	22,143	20096,485	,000	1	,999
	Konstante	-,940	,236	15,885	1	<,001

Variablen in der Gleichung

			95% Konfidenzintervall für EXP(B)	
		Exp(B)	Unterer Wert	Oberer Wert
Schritt 1[a]	DSM_5_kor(1)	4135615597,699	,000	.
	Konstante	,391		

a. In Schritt 1 eingegebene Variablen: DSM_5_kor.

```
Logistische Regression
```

Hinweise

Ausgabe erstellt		11-MAR-2026 18:19:50
Kommentare		
Eingabe	Daten	H:\Gabapentin Auswertung\28.02.24\6.1.2022 Gabapentin_1.sav
	Aktiver Datensatz	DataSet1
	Filter	<keine>
	Gewichtung	<keine>
	Aufgeteilte Datei	<keine>
	Anzahl der Zeilen in der Arbeitsdatei	93
Behandlung fehlender Werte	Definition für "fehlend"	Benutzerdefinierte fehlende Werte werden als fehlend behandelt
Syntax		LOGISTIC REGRESSION VARIABLES SUD1_0 /METHOD=ENTER DSM_6_kor

		/CONTRAST (DSM_6_kor)=Indicator(1) /PRINT=CI(95) /CRITERIA=PIN(0.05) POUT(0.10) ITERATE(20) CUT(0.5).
Ressourcen	Prozessorzeit	00:00:00,00
	Verstrichene Zeit	00:00:00,01

Zusammenfassung der Fallverarbeitung

Ungewichtete Fälle[a]		N	Prozent
Ausgewählte Fälle	Einbezogen in Analyse	93	100,0
	Fehlende Fälle	0	,0
	Gesamt	93	100,0
Nicht ausgewählte Fälle		0	,0
Gesamt		93	100,0

a. Wenn die Gewichtung wirksam ist, finden Sie die Gesamtzahl der Fälle in der Klassifizierungstabelle.

Codierung abhängiger Variablen

Ursprünglicher Wert	Interner Wert
0	0
1	1

Codierungen kategorialer Variablen

		Häufigkeit	Parametercodierung (1)
DSM_6_kor	0	92	,000
	1	1	1,000

Block 0: Anfangsblock

Klassifizierungstabelle[a,b]

Beobachtet			Vorhergesagt SUD1_0: 0	Vorhergesagt SUD1_0: 1	Prozentsatz der Richtigen
Schritt 0	SUD1_0	0	64	0	100,0
		1	29	0	,0
	Gesamtprozentsatz				68,8

a. Konstante in das Modell einbezogen.

b. Der Trennwert lautet ,500

Variablen in der Gleichung

		RegressionskoeffizientB	Standardfehler	Wald	df	Sig.	Exp(B)
Schritt 0	Konstante	-,792	,224	12,505	1	<,001	,453

Variablen nicht in der Gleichung

			Wert	df	Sig.
Schritt 0	Variablen	DSM_6_kor(1)	2,231	1	,135
	Gesamtstatistik		2,231	1	,135

Block 1: Methode = Einschluß

Omnibus-Tests der Modellkoeffizienten

		Chi-Quadrat	df	Sig.
Schritt 1	Schritt	2,355	1	,125
	Block	2,355	1	,125
	Modell	2,355	1	,125

Modellzusammenfassung

Schritt	-2 Log-Likelihood	Cox & Snell R-Quadrat	Nagelkerkes R-Quadrat
1	113,069[a]	,025	,035

a. Schätzung beendet bei Iteration Nummer 20 weil die Höchstzahl der Iterationen erreicht wurde. Endlösung kann nicht gefunden werden.

Klassifizierungstabelle[a]

Beobachtet			Vorhergesagt SUD1_0: 0	Vorhergesagt SUD1_0: 1	Prozentsatz der Richtigen
Schritt 1	SUD1_0	0	64	0	100,0
		1	28	1	3,4
	Gesamtprozentsatz				69,9

a. Der Trennwert lautet ,500

Variablen in der Gleichung

		RegressionskoeffizientB	Standardfehler	Wald	df	Sig.
Schritt 1[a]	DSM_6_kor(1)	22,030	40192,969	,000	1	1,000
	Konstante	-,827	,227	13,311	1	<,001

Variablen in der Gleichung

		Exp(B)	95% Konfidenzintervall für EXP(B): Unterer Wert	95% Konfidenzintervall für EXP(B): Oberer Wert
Schritt 1[a]	DSM_6_kor(1)	3692513926,517	,000	.
	Konstante	,437		

a. In Schritt 1 eingegebene Variablen: DSM_6_kor.

Logistische Regression

Hinweise

Ausgabe erstellt		11-MAR-2026 18:19:50
Kommentare		
Eingabe	Daten	H:\Gabapentin Auswertung\28.02.24\6.1.2022 Gabapentin_1.sav
	Aktiver Datensatz	DataSet1
	Filter	<keine>
	Gewichtung	<keine>
	Aufgeteilte Datei	<keine>
	Anzahl der Zeilen in der Arbeitsdatei	93
Behandlung fehlender Werte	Definition für "fehlend"	Benutzerdefinierte fehlende Werte werden als fehlend behandelt
Syntax		LOGISTIC REGRESSION VARIABLES SUD1_0 /METHOD=ENTER DSM_7_kor /CONTRAST (DSM_7_kor)=Indicator(1) /PRINT=CI(95) /CRITERIA=PIN(0.05) POUT(0.10) ITERATE(20) CUT(0.5).
Ressourcen	Prozessorzeit	00:00:00,02
	Verstrichene Zeit	00:00:00,01

Zusammenfassung der Fallverarbeitung

Ungewichtete Fälle[a]		N	Prozent
Ausgewählte Fälle	Einbezogen in Analyse	93	100,0
	Fehlende Fälle	0	,0
	Gesamt	93	100,0
Nicht ausgewählte Fälle		0	,0
Gesamt		93	100,0

a. Wenn die Gewichtung wirksam ist, finden Sie die Gesamtzahl der Fälle in der Klassifizierungstabelle.

Codierung abhängiger Variablen

Ursprünglicher Wert	Interner Wert
0	0
1	1

Codierungen kategorialer Variablen

		Häufigkeit	Parametercodierung (1)
DSM_7_kor	0	81	,000
	1	12	1,000

```
Block 0: Anfangsblock
```

Klassifizierungstabelle[a,b]

	Beobachtet		Vorhergesagt SUD1_0 0	Vorhergesagt SUD1_0 1	Prozentsatz der Richtigen
Schritt 0	SUD1_0	0	64	0	100,0
		1	29	0	,0
	Gesamtprozentsatz				68,8

a. Konstante in das Modell einbezogen.

b. Der Trennwert lautet ,500

Variablen in der Gleichung

		RegressionskoeffizientB	Standardfehler	Wald	df	Sig.	Exp(B)
Schritt 0	Konstante	-,792	,224	12,505	1	<,001	,453

Variablen nicht in der Gleichung

			Wert	df	Sig.
Schritt 0	Variablen	DSM_7_kor(1)	30,406	1	<,001
	Gesamtstatistik		30,406	1	<,001

```
Block 1: Methode = Einschluß
```

Omnibus-Tests der Modellkoeffizienten

		Chi-Quadrat	df	Sig.
Schritt 1	Schritt	32,189	1	<,001
	Block	32,189	1	<,001
	Modell	32,189	1	<,001

Modellzusammenfassung

Schritt	-2 Log-Likelihood	Cox & Snell R-Quadrat	Nagelkerkes R-Quadrat
1	83,234[a]	,293	,412

a. Schätzung beendet bei Iteration Nummer 20 weil die Höchstzahl der Iterationen erreicht wurde. Endlösung kann nicht gefunden werden.

Klassifizierungstabelle[a]

		Vorhergesagt		
		SUD1_0		Prozentsatz der Richtigen
Beobachtet		0	1	

Schritt 1	SUD1_0	0	64	0	100,0
		1	17	12	41,4
	Gesamtprozentsatz				81,7

a. Der Trennwert lautet ,500

Variablen in der Gleichung

		RegressionskoeffizientB	Standardfehler	Wald	df	Sig.
Schritt 1[a]	DSM_7_kor(1)	22,529	11602,711	,000	1	,998
	Konstante	-1,326	,273	23,606	1	<,001

Variablen in der Gleichung

		Exp(B)	95% Konfidenzintervall für EXP(B) Unterer Wert	Oberer Wert
Schritt 1[a]	DSM_7_kor(1)	6081787643,675	,000	.
	Konstante	,266		

a. In Schritt 1 eingegebene Variablen: DSM_7_kor.

```
Logistische Regression
```

Hinweise

Ausgabe erstellt		11-MAR-2026 18:19:50
Kommentare		
Eingabe	Daten	H:\Gabapentin Auswertung\28.02.24\6.1.2022 Gabapentin_1.sav

	Aktiver Datensatz	DataSet1
	Filter	<keine>
	Gewichtung	<keine>
	Aufgeteilte Datei	<keine>
	Anzahl der Zeilen in der Arbeitsdatei	93
Behandlung fehlender Werte	Definition für "fehlend"	Benutzerdefinierte fehlende Werte werden als fehlend behandelt
Syntax		LOGISTIC REGRESSION VARIABLES SUD1_0 /METHOD=ENTER DSM_8_kor /CONTRAST (DSM_8_kor)=Indicator(1) /PRINT=CI(95) /CRITERIA=PIN(0.05) POUT(0.10) ITERATE(20) CUT(0.5).
Ressourcen	Prozessorzeit	00:00:00,00
	Verstrichene Zeit	00:00:00,01

Zusammenfassung der Fallverarbeitung

Ungewichtete Fälle[a]		N	Prozent
Ausgewählte Fälle	Einbezogen in Analyse	93	100,0
	Fehlende Fälle	0	,0
	Gesamt	93	100,0
Nicht ausgewählte Fälle		0	,0
Gesamt		93	100,0

a. Wenn die Gewichtung wirksam ist, finden Sie die Gesamtzahl der Fälle in der Klassifizierungstabelle.

Codierung abhängiger Variablen

Ursprünglicher	Interner

Wert	Wert
0	0
1	1

Codierungen kategorialer Variablen

		Häufigkeit	Parametercodierung (1)
DSM_8_kor	0	87	,000
	1	6	1,000

```
Block 0: Anfangsblock
```

Klassifizierungstabelle[a,b]

Beobachtet			Vorhergesagt SUD1_0 0	Vorhergesagt SUD1_0 1	Prozentsatz der Richtigen
Schritt 0	SUD1_0	0	64	0	100,0
		1	29	0	,0
	Gesamtprozentsatz				68,8

a. Konstante in das Modell einbezogen.

b. Der Trennwert lautet ,500

Variablen in der Gleichung

		RegressionskoeffizientB	Standardfehler	Wald	df	Sig.	Exp(B)
Schritt 0	Konstante	-,792	,224	12,505	1	<,001	,453

Variablen nicht in der Gleichung

			Wert	df	Sig.
Schritt 0	Variablen	DSM_8_kor(1)	8,129	1	,004
	Gesamtstatistik		8,129	1	,004

Block 1: Methode = Einschluß

Omnibus-Tests der Modellkoeffizienten

		Chi-Quadrat	df	Sig.
Schritt 1	Schritt	7,530	1	,006
	Block	7,530	1	,006
	Modell	7,530	1	,006

Modellzusammenfassung

Schritt	-2 Log-Likelihood	Cox & Snell R-Quadrat	Nagelkerkes R-Quadrat
1	107,893[a]	,078	,109

a. Schätzung beendet bei Iteration Nummer 4, weil die Parameterschätzer sich um weniger als ,001 änderten.

Klassifizierungstabelle[a]

Beobachtet			Vorhergesagt SUD1_0: 0	Vorhergesagt SUD1_0: 1	Prozentsatz der Richtigen
Schritt 1	SUD1_0	0	63	1	98,4
		1	24	5	17,2
	Gesamtprozentsatz				73,1

a. Der Trennwert lautet ,500

Variablen in der Gleichung

		RegressionskoeffizientB	Standardfehler	Wald	df	Sig.
Schritt 1[a]	DSM_8_kor(1)	2,575	1,121	5,271	1	,022
	Konstante	-,965	,240	16,187	1	<,001

Variablen in der Gleichung

			95% Konfidenzintervall für EXP(B)	
		Exp(B)	Unterer Wert	Oberer Wert
Schritt 1[a]	DSM_8_kor(1)	13,125	1,457	118,205
	Konstante	,381		

a. In Schritt 1 eingegebene Variablen: DSM_8_kor.

```
Logistische Regression
```

Hinweise

Ausgabe erstellt		11-MAR-2026 18:19:51
Kommentare		
Eingabe	Daten	H:\Gabapentin Auswertung\28.02.24\6.1.2022 Gabapentin_1.sav
	Aktiver Datensatz	DataSet1
	Filter	<keine>
	Gewichtung	<keine>
	Aufgeteilte Datei	<keine>
	Anzahl der Zeilen in der Arbeitsdatei	93
Behandlung fehlender Werte	Definition für "fehlend"	Benutzerdefinierte fehlende Werte werden als fehlend behandelt
Syntax		LOGISTIC REGRESSION VARIABLES SUD1_0 /METHOD=ENTER DSM_9_kor

		/CONTRAST (DSM_9_kor)=Indicator(1) /PRINT=CI(95) /CRITER
Ressourcen	Prozessorzeit	00:00:00,00
	Verstrichene Zeit	00:00:00,00

Warnungen

Keine Schlüsselwörter im Unterbefehl CRITERIA gefunden. Standardwerte werden verwendet.

Zusammenfassung der Fallverarbeitung

Ungewichtete Fälle[a]		N	Prozent
Ausgewählte Fälle	Einbezogen in Analyse	93	100,0
	Fehlende Fälle	0	,0
	Gesamt	93	100,0
Nicht ausgewählte Fälle		0	,0
Gesamt		93	100,0

a. Wenn die Gewichtung wirksam ist, finden Sie die Gesamtzahl der Fälle in der Klassifizierungstabelle.

Codierung abhängiger Variablen

Ursprünglicher Wert	Interner Wert
0	0
1	1

Codierungen kategorialer Variablen

		Häufigkeit	Parametercodierung (1)
DSM_9_kor	0	78	,000
	1	15	1,000

Block 0: Anfangsblock

Klassifizierungstabelle[a,b]

			Vorhergesagt		
			SUD1_0		Prozentsatz der Richtigen
Beobachtet			0	1	
Schritt 0	SUD1_0	0	64	0	100,0
		1	29	0	,0
	Gesamtprozentsatz				68,8

a. Konstante in das Modell einbezogen.

b. Der Trennwert lautet ,500

Variablen in der Gleichung

		RegressionskoeffizientB	Standardfehler	Wald	df	Sig.	Exp(B)
Schritt 0	Konstante	-,792	,224	12,505	1	<,001	,453

Variablen nicht in der Gleichung

			Wert	df	Sig.
Schritt 0	Variablen	DSM_9_kor(1)	32,193	1	<,001
	Gesamtstatistik		32,193	1	<,001

Block 1: Methode = Einschluß

Omnibus-Tests der Modellkoeffizienten

		Chi-Quadrat	df	Sig.
Schritt 1	Schritt	31,705	1	<,001
	Block	31,705	1	<,001
	Modell	31,705	1	<,001

Modellzusammenfassung

Schritt	-2 Log-Likelihood	Cox & Snell R-Quadrat	Nagelkerkes R-Quadrat
1	83,718[a]	,289	,406

a. Schätzung beendet bei Iteration Nummer 5, weil die Parameterschätzer sich um weniger als ,001 änderten.

Klassifizierungstabelle[a]

	Beobachtet		Vorhergesagt SUD1_0: 0	Vorhergesagt SUD1_0: 1	Prozentsatz der Richtigen
Schritt 1	SUD1_0	0	63	1	98,4
		1	15	14	48,3
	Gesamtprozentsatz				82,8

a. Der Trennwert lautet ,500

Variablen in der Gleichung

		RegressionskoeffizientB	Standardfehler	Wald	df	Sig.
Schritt 1[a]	DSM_9_kor(1)	4,074	1,074	14,384	1	<,001
	Konstante	-1,435	,287	24,951	1	<,001

Variablen in der Gleichung

		Exp(B)	95% Konfidenzintervall für EXP(B): Unterer Wert	95% Konfidenzintervall für EXP(B): Oberer Wert
Schritt 1[a]	DSM_9_kor(1)	58,800	7,161	482,795
	Konstante	,238		

a. In Schritt 1 eingegebene Variablen: DSM_9_kor.

Logistische Regression

Hinweise

Ausgabe erstellt		11-MAR-2026 18:19:51
Kommentare		
Eingabe	Daten	H:\Gabapentin Auswertung\28.02.24\6.1.2022 Gabapentin_1.sav
	Aktiver Datensatz	DataSet1
	Filter	<keine>
	Gewichtung	<keine>
	Aufgeteilte Datei	<keine>
	Anzahl der Zeilen in der Arbeitsdatei	93
Behandlung fehlender Werte	Definition für "fehlend"	Benutzerdefinierte fehlende Werte werden als fehlend behandelt
Syntax		LOGISTIC REGRESSION VARIABLES SUD1_0 /METHOD=ENTER DSM_10_kor /CONTRAST (DSM_10_kor)=Indicator (1) /PRINT=CI(95) /CRITERIA=PIN(0.05) POUT(0.10) ITERATE(20) CUT(0.5).IA=PIN(0.05) POUT(0.10) ITERATE(20) CUT(0.5).
Ressourcen	Prozessorzeit	00:00:00,00

Verstrichene Zeit	00:00:00,00

Warnungen

Ungültiges Schlüsselwort im Unterbefehl CRITERIA. Die gültigen Schlüsselwörter lauten: BCON, PCON, ITERATE, LCON, PIN, POUT, EPSILON, CUT.
Die Ausführung dieses Befehls wurde unterbrochen.

Logistische Regression

Hinweise

Ausgabe erstellt		11-MAR-2026 18:19:51
Kommentare		
Eingabe	Daten	H:\Gabapentin Auswertung\28.02.24\6.1.2022 Gabapentin_1.sav
	Aktiver Datensatz	DataSet1
	Filter	<keine>
	Gewichtung	<keine>
	Aufgeteilte Datei	<keine>
	Anzahl der Zeilen in der Arbeitsdatei	93
Behandlung fehlender Werte	Definition für "fehlend"	Benutzerdefinierte fehlende Werte werden als fehlend behandelt
Syntax		LOGISTIC REGRESSION VARIABLES SUD1_0 /METHOD=ENTER DSM_11_kor /CONTRAST (DSM_11_kor)=Indicator (1) /PRINT=CI(95) /CRITERIA=PIN(0.05) POUT(0.10)

		ITERATE(20) CUT(0.5).
Ressourcen	Prozessorzeit	00:00:00,02
	Verstrichene Zeit	00:00:00,01

Zusammenfassung der Fallverarbeitung

Ungewichtete Fälle[a]		N	Prozent
Ausgewählte Fälle	Einbezogen in Analyse	93	100,0
	Fehlende Fälle	0	,0
	Gesamt	93	100,0
Nicht ausgewählte Fälle		0	,0
Gesamt		93	100,0

a. Wenn die Gewichtung wirksam ist, finden Sie die Gesamtzahl der Fälle in der Klassifizierungstabelle.

Codierung abhängiger Variablen

Ursprünglicher Wert	Interner Wert
0	0
1	1

Codierungen kategorialer Variablen

		Häufigkeit	Parametercodierung (1)
DSM_11_kor	0	77	,000
	1	16	1,000

```
Block 0: Anfangsblock
```

Klassifizierungstabelle[a,b]

Beobachtet Vorhergesagt

			SUD1_0		Prozentsatz der Richtigen
			0	1	
Schritt 0	SUD1_0	0	64	0	100,0
		1	29	0	,0
	Gesamtprozentsatz				68,8

a. Konstante in das Modell einbezogen.

b. Der Trennwert lautet ,500

Variablen in der Gleichung

		RegressionskoeffizientB	Standardfehler	Wald	df	Sig.	Exp(B)
Schritt 0	Konstante	-,792	,224	12,505	1	<,001	,453

Variablen nicht in der Gleichung

			Wert	df	Sig.
Schritt 0	Variablen	DSM_11_kor(1)	5,659	1	,017
	Gesamtstatistik		5,659	1	,017

```
Block 1: Methode = Einschluß
```

Omnibus-Tests der Modellkoeffizienten

		Chi-Quadrat	df	Sig.
Schritt 1	Schritt	5,284	1	,022
	Block	5,284	1	,022
	Modell	5,284	1	,022

Modellzusammenfassung

Schritt	-2 Log-Likelihood	Cox & Snell R-Quadrat	Nagelkerkes R-Quadrat
1	110,139[a]	,055	,078

a. Schätzung beendet bei Iteration Nummer 4, weil die

Parameterschätzer sich um weniger als ,001 änderten.

Klassifizierungstabelle[a]

			Vorhergesagt		
			SUD1_0		Prozentsatz der Richtigen
	Beobachtet		0	1	
Schritt 1	SUD1_0	0	57	7	89,1
		1	20	9	31,0
	Gesamtprozentsatz				71,0

a. Der Trennwert lautet ,500

Variablen in der Gleichung

		RegressionskoeffizientB	Standardfehler	Wald	df	Sig.
Schritt 1[a]	DSM_11_kor(1)	1,299	,567	5,245	1	,022
	Konstante	-1,047	,260	16,239	1	<,001

Variablen in der Gleichung

		Exp(B)	95% Konfidenzintervall für EXP(B)	
			Unterer Wert	Oberer Wert
Schritt 1[a]	DSM_11_kor(1)	3,664	1,206	11,134
	Konstante	,351		

a. In Schritt 1 eingegebene Variablen: DSM_11_kor.

Logistische Regression

Hinweise

Ausgabe erstellt		11-MAR-2026 18:19:51
Kommentare		
Eingabe	Daten	H:\Gabapentin Auswertung\28.02.24\6.1.2022 Gabapentin_1.sav
	Aktiver Datensatz	DataSet1
	Filter	<keine>
	Gewichtung	<keine>
	Aufgeteilte Datei	<keine>
	Anzahl der Zeilen in der Arbeitsdatei	93
Behandlung fehlender Werte	Definition für "fehlend"	Benutzerdefinierte fehlende Werte werden als fehlend behandelt
Syntax		LOGISTIC REGRESSION VARIABLES SUD1_0 /METHOD=ENTER DEQ_1_korr /CONTRAST (DEQ_1_korr)=Indicator(1) /PRINT=CI(95) /CRITERIA=PIN(0.05) POUT(0.10) ITERATE(20) CUT(0.5).
Ressourcen	Prozessorzeit	00:00:00,02
	Verstrichene Zeit	00:00:00,01

Zusammenfassung der Fallverarbeitung

Ungewichtete Fälle[a]		N	Prozent
Ausgewählte Fälle	Einbezogen in Analyse	93	100,0
	Fehlende Fälle	0	,0
	Gesamt	93	100,0
Nicht ausgewählte Fälle		0	,0
Gesamt		93	100,0

a. Wenn die Gewichtung wirksam ist, finden Sie die Gesamtzahl der Fälle in der Klassifizierungstabelle.

Codierung abhängiger Variablen

Ursprünglicher Wert	Interner Wert
0	0
1	1

Codierungen kategorialer Variablen

		Häufigkeit	Parametercodierung (1)	(2)	(3)	(4)	(5)
DEQ_1_korr	1	11	,000	,000	,000	,000	,000
	2	5	1,000	,000	,000	,000	,000
	3	1	,000	1,000	,000	,000	,000
	4	1	,000	,000	1,000	,000	,000
	5	6	,000	,000	,000	1,000	,000
	6	2	,000	,000	,000	,000	1,000
	7	2	,000	,000	,000	,000	,000
	8	5	,000	,000	,000	,000	,000
	9	5	,000	,000	,000	,000	,000
	10	55	,000	,000	,000	,000	,000

Codierungen kategorialer Variablen

		Parametercodierung (6)	(7)	(8)	(9)
DEQ_1_korr	1	,000	,000	,000	,000
	2	,000	,000	,000	,000
	3	,000	,000	,000	,000
	4	,000	,000	,000	,000
	5	,000	,000	,000	,000
	6	,000	,000	,000	,000
	7	1,000	,000	,000	,000
	8	,000	1,000	,000	,000
	9	,000	,000	1,000	,000
	10	,000	,000	,000	1,000

Block 0: Anfangsblock

Klassifizierungstabelle[a,b]

Beobachtet			Vorhergesagt SUD1_0 0	1	Prozentsatz der Richtigen
Schritt 0	SUD1_0	0	64	0	100,0
		1	29	0	,0
	Gesamtprozentsatz				68,8

a. Konstante in das Modell einbezogen.

b. Der Trennwert lautet ,500

Variablen in der Gleichung

		RegressionskoeffizientB	Standardfehler	Wald	df	Sig.	Exp(B)
Schritt 0	Konstante	-,792	,224	12,505	1	<,001	,453

Variablen nicht in der Gleichung

			Wert	df	Sig.
Schritt 0	Variablen	DEQ_1_korr	10,857	9	,286
		DEQ_1_korr(1)	2,394	1	,122
		DEQ_1_korr(2)	,458	1	,499
		DEQ_1_korr(3)	2,231	1	,135
		DEQ_1_korr(4)	1,058	1	,304
		DEQ_1_korr(5)	,337	1	,561
		DEQ_1_korr(6)	,337	1	,561
		DEQ_1_korr(7	2,394	1	,122

	)			
	DEQ_1_korr(8)	2,045	1	,153
	DEQ_1_korr(9)	,005	1	,945
	Gesamtstatistik	10,857	9	,286

```
Block 1: Methode = Einschluß
```

Omnibus-Tests der Modellkoeffizienten

		Chi-Quadrat	df	Sig.
Schritt 1	Schritt	13,918	9	,125
	Block	13,918	9	,125
	Modell	13,918	9	,125

Modellzusammenfassung

Schritt	-2 Log-Likelihood	Cox & Snell R-Quadrat	Nagelkerkes R-Quadrat
1	101,505[a]	,139	,196

a. Schätzung beendet bei Iteration Nummer 20 weil die Höchstzahl der Iterationen erreicht wurde. Endlösung kann nicht gefunden werden.

Klassifizierungstabelle[a]

	Beobachtet		Vorhergesagt SUD1_0: 0	Vorhergesagt SUD1_0: 1	Prozentsatz der Richtigen
Schritt 1	SUD1_0	0	57	7	89,1
		1	20	9	31,0
	Gesamtprozentsatz				71,0

a. Der Trennwert lautet ,500

Variablen in der Gleichung

		Regressionskoeffizient B	Standardfehler	Wald	df	Sig.
Schritt 1[a]	DEQ_1_korr			2,905	9	,968
	DEQ_1_korr(1)	-20,222	17974,843	,000	1	,999
	DEQ_1_korr(2)	-20,222	40192,970	,000	1	1,000
	DEQ_1_korr(3)	22,184	40192,969	,000	1	1,000
	DEQ_1_korr(4)	,981	1,061	,855	1	,355
	DEQ_1_korr(5)	,981	1,568	,391	1	,532
	DEQ_1_korr(6)	,981	1,568	,391	1	,532
	DEQ_1_korr(7)	-20,222	17974,843	,000	1	,999
	DEQ_1_korr(8)	1,386	1,137	1,488	1	,223
	DEQ_1_korr(9)	,176	,737	,057	1	,811
	Konstante	-,981	,677	2,099	1	,147

Variablen in der Gleichung

		Exp(B)	95% Konfidenzintervall für EXP(B)	
			Unterer Wert	Oberer Wert
Schritt 1[a]	DEQ_1_korr			
	DEQ_1_korr(1)	,000	,000	.
	DEQ_1_korr(2)	,000	,000	.
	DEQ_1_korr(3)	4307932914,270	,000	.
	DEQ_1_korr(4)	2,667	,334	21,321
	DEQ_1_korr(5)	2,667	,123	57,620

	DEQ_1_korr(6)	2,667	,123	57,620
	DEQ_1_korr(7)	,000	,000	.
	DEQ_1_korr(8)	4,000	,431	37,108
	DEQ_1_korr(9)	1,193	,281	5,060
	Konstante	,375		

a. In Schritt 1 eingegebene Variablen: DEQ_1_korr.

Logistische Regression

Hinweise

Ausgabe erstellt		11-MAR-2026 18:19:51
Kommentare		
Eingabe	Daten	H:\Gabapentin Auswertung\28.02.24\6.1.2022 Gabapentin_1.sav
	Aktiver Datensatz	DataSet1
	Filter	<keine>
	Gewichtung	<keine>
	Aufgeteilte Datei	<keine>
	Anzahl der Zeilen in der Arbeitsdatei	93
Behandlung fehlender Werte	Definition für "fehlend"	Benutzerdefinierte fehlende Werte werden als fehlend behandelt
Syntax		LOGISTIC REGRESSION VARIABLES SUD1_0 /METHOD=ENTER DEQ_2_korr

		/CONTRAST (DEQ_2_korr)=Indicator(1) /PRINT=CI(95) /CRITERIA=PIN(0.05) POUT(0.10) ITERATE(20) CUT(0.5).
Ressourcen	Prozessorzeit	00:00:00,00
	Verstrichene Zeit	00:00:00,01

Zusammenfassung der Fallverarbeitung

Ungewichtete Fälle[a]		N	Prozent
Ausgewählte Fälle	Einbezogen in Analyse	93	100,0
	Fehlende Fälle	0	,0
	Gesamt	93	100,0
Nicht ausgewählte Fälle		0	,0
Gesamt		93	100,0

a. Wenn die Gewichtung wirksam ist, finden Sie die Gesamtzahl der Fälle in der Klassifizierungstabelle.

Codierung abhängiger Variablen

Ursprünglicher Wert	Interner Wert
0	0
1	1

Codierungen kategorialer Variablen

		Häufigkeit	Parametercodierung				
			(1)	(2)	(3)	(4)	(5)
DEQ_2_korr	1	62	,000	,000	,000	,000	,000
	2	7	1,000	,000	,000	,000	,000
	3	2	,000	1,000	,000	,000	,000
	5	5	,000	,000	1,000	,000	,000
	7	3	,000	,000	,000	1,000	,000
	8	4	,000	,000	,000	,000	1,000

	9	2	,000	,000	,000	,000	,000
	10	8	,000	,000	,000	,000	,000

Codierungen kategorialer Variablen

		Parametercodierung	
		(6)	(7)
DEQ_2_korr	1	,000	,000
	2	,000	,000
	3	,000	,000
	5	,000	,000
	7	,000	,000
	8	,000	,000
	9	1,000	,000
	10	,000	1,000

Block 0: Anfangsblock

Klassifizierungstabelle[a,b]

Beobachtet			Vorhergesagt SUD1_0 0	1	Prozentsatz der Richtigen
Schritt 0	SUD1_0	0	64	0	100,0
		1	29	0	,0
	Gesamtprozentsatz				68,8

a. Konstante in das Modell einbezogen.

b. Der Trennwert lautet ,500

Variablen in der Gleichung

		RegressionskoeffizientB	Standardfehler	Wald	df	Sig.	Exp(B)
Schritt 0	Konstante	-,792	,224	12,505	1	<,001	,453

Variablen nicht in der Gleichung

			Wert	df	Sig.
Schritt 0	Variablen	DEQ_2_korr	4,920	7	,670
		DEQ_2_korr(1)	1,007	1	,316
		DEQ_2_korr(2)	,337	1	,561
		DEQ_2_korr(3)	,191	1	,662
		DEQ_2_korr(4)	,007	1	,935
		DEQ_2_korr(5)	1,894	1	,169
		DEQ_2_korr(6)	,337	1	,561
		DEQ_2_korr(7)	1,444	1	,229
	Gesamtstatistik		4,920	7	,670

```
Block 1: Methode = Einschluß
```

Omnibus-Tests der Modellkoeffizienten

		Chi-Quadrat	df	Sig.
Schritt 1	Schritt	6,084	7	,530
	Block	6,084	7	,530
	Modell	6,084	7	,530

Modellzusammenfassung

Schritt	-2 Log-Likelihood	Cox & Snell R-Quadrat	Nagelkerkes R-Quadrat
1	109,339[a]	,063	,089

a. Schätzung beendet bei Iteration Nummer 20 weil die Höchstzahl der Iterationen erreicht wurde. Endlösung kann nicht gefunden werden.

Klassifizierungstabelle[a]

Beobachtet			Vorhergesagt SUD1_0: 0	Vorhergesagt SUD1_0: 1	Prozentsatz der Richtigen
Schritt 1	SUD1_0	0	58	6	90,6
		1	23	6	20,7
	Gesamtprozentsatz				68,8

a. Der Trennwert lautet ,500

Variablen in der Gleichung

		RegressionskoeffizientB	Standardfehler	Wald	df	Sig.
Schritt 1[a]	DEQ_2_korr			2,775	7	,905
	DEQ_2_korr(1)	-,975	1,115	,765	1	,382
	DEQ_2_korr(2)	,817	1,441	,321	1	,571
	DEQ_2_korr(3)	,411	,954	,186	1	,666
	DEQ_2_korr(4)	,124	1,255	,010	1	,922
	DEQ_2_korr(5)	-20,386	20096,485	,000	1	,999
	DEQ_2_korr(6)	,817	1,441	,321	1	,571
	DEQ_2_korr(7)	,817	,759	1,158	1	,282
	Konstante	-,817	,275	8,791	1	,003

Variablen in der Gleichung

		Exp(B)	95% Konfidenzintervall für EXP(B): Unterer Wert	95% Konfidenzintervall für EXP(B): Oberer Wert
Schritt 1[a]	DEQ_2_korr			
	DEQ_2_korr(1)	,377	,042	3,353

	DEQ_2_korr(2)	2,263	,134	38,117
	DEQ_2_korr(3)	1,509	,233	9,778
	DEQ_2_korr(4)	1,132	,097	13,251
	DEQ_2_korr(5)	,000	,000	.
	DEQ_2_korr(6)	2,263	,134	38,117
	DEQ_2_korr(7)	2,263	,511	10,015
	Konstante	,442		

a. In Schritt 1 eingegebene Variablen: DEQ_2_korr.

```
Logistische Regression
```

Hinweise

Ausgabe erstellt		11-MAR-2026 18:19:51
Kommentare		
Eingabe	Daten	H:\Gabapentin Auswertung\28.02.24\6.1.2022 Gabapentin_1.sav
	Aktiver Datensatz	DataSet1
	Filter	<keine>
	Gewichtung	<keine>
	Aufgeteilte Datei	<keine>
	Anzahl der Zeilen in der Arbeitsdatei	93
Behandlung fehlender Werte	Definition für "fehlend"	Benutzerdefinierte fehlende Werte werden als fehlend behandelt

Syntax		LOGISTIC REGRESSION VARIABLES SUD1_0 /METHOD=ENTER DEQ_3_korr /CONTRAST (DEQ_3_korr)=Indicator(1) /PRINT=CI(95) /CRITERIA=PIN(0.05) POUT(0.10) ITERATE(20) CUT(0.5).
Ressourcen	Prozessorzeit	00:00:00,00
	Verstrichene Zeit	00:00:00,01

Zusammenfassung der Fallverarbeitung

Ungewichtete Fälle[a]		N	Prozent
Ausgewählte Fälle	Einbezogen in Analyse	93	100,0
	Fehlende Fälle	0	,0
	Gesamt	93	100,0
Nicht ausgewählte Fälle		0	,0
Gesamt		93	100,0

a. Wenn die Gewichtung wirksam ist, finden Sie die Gesamtzahl der Fälle in der Klassifizierungstabelle.

Codierung abhängiger Variablen

Ursprünglicher Wert	Interner Wert
0	0
1	1

Codierungen kategorialer Variablen

			Parametercodierung				
		Häufigkeit	(1)	(2)	(3)	(4)	(5)
DEQ_3_korr	1	14	,000	,000	,000	,000	,000
	2	5	1,000	,000	,000	,000	,000

	3	3	,000	1,000	,000	,000	,000
	4	2	,000	,000	1,000	,000	,000
	5	3	,000	,000	,000	1,000	,000
	6	2	,000	,000	,000	,000	1,000
	7	2	,000	,000	,000	,000	,000
	8	13	,000	,000	,000	,000	,000
	9	4	,000	,000	,000	,000	,000
	10	45	,000	,000	,000	,000	,000

Codierungen kategorialer Variablen

		Parametercodierung			
		(6)	(7)	(8)	(9)
DEQ_3_korr	1	,000	,000	,000	,000
	2	,000	,000	,000	,000
	3	,000	,000	,000	,000
	4	,000	,000	,000	,000
	5	,000	,000	,000	,000
	6	,000	,000	,000	,000
	7	1,000	,000	,000	,000
	8	,000	1,000	,000	,000
	9	,000	,000	1,000	,000
	10	,000	,000	,000	1,000

Block 0: Anfangsblock

Klassifizierungstabelle[a,b]

			Vorhergesagt		
			SUD1_0		Prozentsatz der Richtigen
	Beobachtet		0	1	
Schritt 0	SUD1_0	0	64	0	100,0
		1	29	0	,0
	Gesamtprozentsatz				68,8

a. Konstante in das Modell einbezogen.

b. Der Trennwert lautet ,500

Variablen in der Gleichung

		Regressionskoeffizient B	Standardfehler	Wald	df	Sig.	Exp(B)
Schritt 0	Konstante	-,792	,224	12,505	1	<,001	,453

Variablen nicht in der Gleichung

			Wert	df	Sig.
Schritt 0	Variablen	DEQ_3_korr	9,790	9	,368
		DEQ_3_korr(1)	2,394	1	,122
		DEQ_3_korr(2)	1,405	1	,236
		DEQ_3_korr(3)	,926	1	,336
		DEQ_3_korr(4)	,007	1	,935
		DEQ_3_korr(5)	,337	1	,561
		DEQ_3_korr(6)	,926	1	,336
		DEQ_3_korr(7)	3,617	1	,057
		DEQ_3_korr(8)	,690	1	,406
		DEQ_3_korr(9)	,214	1	,644
	Gesamtstatistik		9,790	9	,368

```
Block 1: Methode = Einschluß
```

Omnibus-Tests der Modellkoeffizienten

		Chi-Quadrat	df	Sig.
Schritt 1	Schritt	12,989	9	,163
	Block	12,989	9	,163

	Modell	12,989	9	,163

Modellzusammenfassung

Schritt	-2 Log-Likelihood	Cox & Snell R-Quadrat	Nagelkerkes R-Quadrat
1	102,435[a]	,130	,183

a. Schätzung beendet bei Iteration Nummer 20 weil die Höchstzahl der Iterationen erreicht wurde. Endlösung kann nicht gefunden werden.

Klassifizierungstabelle[a]

Beobachtet			Vorhergesagt SUD1_0 0	1	Prozentsatz der Richtigen
Schritt 1	SUD1_0	0	55	9	85,9
		1	19	10	34,5
	Gesamtprozentsatz				69,9

a. Der Trennwert lautet ,500

Variablen in der Gleichung

		RegressionskoeffizientB	Standardfehler	Wald	df	Sig.
Schritt 1[a]	DEQ_3_korr			3,204	9	,956
	DEQ_3_korr(1)	-20,615	17974,843	,000	1	,999
	DEQ_3_korr(2)	-20,615	23205,422	,000	1	,999
	DEQ_3_korr(3)	-20,615	28420,722	,000	1	,999
	DEQ_3_korr(4)	-,105	1,346	,006	1	,938
	DEQ_3_korr(5)	,588	1,520	,149	1	,699
	DEQ_3_korr(6)	-20,615	28420,722	,000	1	,999
	DEQ_3_korr(7	,742	,788	,887	1	,346

	)					
	DEQ_3_korr(8)	,588	1,145	,264	1	,608
	DEQ_3_korr(9)	-,313	,648	,234	1	,629
	Konstante	-,588	,558	1,111	1	,292

Variablen in der Gleichung

		Exp(B)	95% Konfidenzintervall für EXP(B)	
			Unterer Wert	Oberer Wert
Schritt 1[a]	DEQ_3_korr			
	DEQ_3_korr(1)	,000	,000	.
	DEQ_3_korr(2)	,000	,000	.
	DEQ_3_korr(3)	,000	,000	.
	DEQ_3_korr(4)	,900	,064	12,583
	DEQ_3_korr(5)	1,800	,091	35,424
	DEQ_3_korr(6)	,000	,000	.
	DEQ_3_korr(7)	2,100	,448	9,836
	DEQ_3_korr(8)	1,800	,191	16,980
	DEQ_3_korr(9)	,731	,206	2,602
	Konstante	,556		

a. In Schritt 1 eingegebene Variablen: DEQ_3_korr.

Logistische Regression

Hinweise

Ausgabe erstellt		11-MAR-2026 18:19:51
Kommentare		
Eingabe	Daten	H:\Gabapentin Auswertung\28.02.24\6.1.2022 Gabapentin_1.sav
	Aktiver Datensatz	DataSet1
	Filter	<keine>
	Gewichtung	<keine>
	Aufgeteilte Datei	<keine>
	Anzahl der Zeilen in der Arbeitsdatei	93
Behandlung fehlender Werte	Definition für "fehlend"	Benutzerdefinierte fehlende Werte werden als fehlend behandelt
Syntax		LOGISTIC REGRESSION VARIABLES SUD1_0 /METHOD=ENTER DEQ_4_korr /CONTRAST (DEQ_4_korr)=Indicator(1) /PRINT=CI(95) /CRITERIA=PIN(0.05) POUT(0.10) ITERATE(20) CUT(0.5).
Ressourcen	Prozessorzeit	00:00:00,00
	Verstrichene Zeit	00:00:00,01

Zusammenfassung der Fallverarbeitung

Ungewichtete Fälle[a]		N	Prozent
Ausgewählte Fälle	Einbezogen in Analyse	93	100,0
	Fehlende Fälle	0	,0
	Gesamt	93	100,0
Nicht ausgewählte Fälle		0	,0

Gesamt	93	100,0

a. Wenn die Gewichtung wirksam ist, finden Sie die Gesamtzahl der Fälle in der Klassifizierungstabelle.

Codierung abhängiger Variablen

Ursprünglicher Wert	Interner Wert
0	0
1	1

Codierungen kategorialer Variablen

			Parametercodierung				
		Häufigkeit	(1)	(2)	(3)	(4)	(5)
DEQ_4_korr	1	70	,000	,000	,000	,000	,000
	2	3	1,000	,000	,000	,000	,000
	3	4	,000	1,000	,000	,000	,000
	4	1	,000	,000	1,000	,000	,000
	5	1	,000	,000	,000	1,000	,000
	6	4	,000	,000	,000	,000	1,000
	7	2	,000	,000	,000	,000	,000
	8	4	,000	,000	,000	,000	,000
	10	4	,000	,000	,000	,000	,000

Codierungen kategorialer Variablen

		Parametercodierung		
		(6)	(7)	(8)
DEQ_4_korr	1	,000	,000	,000
	2	,000	,000	,000
	3	,000	,000	,000
	4	,000	,000	,000
	5	,000	,000	,000
	6	,000	,000	,000
	7	1,000	,000	,000
	8	,000	1,000	,000
	10	,000	,000	1,000

Block 0: Anfangsblock

Klassifizierungstabelle[a,b]

			Vorhergesagt		
			SUD1_0		Prozentsatz der Richtigen
Beobachtet			0	1	
Schritt 0	SUD1_0	0	64	0	100,0
		1	29	0	,0
	Gesamtprozentsatz				68,8

a. Konstante in das Modell einbezogen.

b. Der Trennwert lautet ,500

Variablen in der Gleichung

		RegressionskoeffizientB	Standardfehler	Wald	df	Sig.	Exp(B)
Schritt 0	Konstante	-,792	,224	12,505	1	<,001	,453

Variablen nicht in der Gleichung

			Wert	df	Sig.
Schritt 0	Variablen	DEQ_4_korr	16,842	8	,032
		DEQ_4_korr(1)	1,405	1	,236
		DEQ_4_korr(2)	1,894	1	,169
		DEQ_4_korr(3)	2,231	1	,135
		DEQ_4_korr(4)	2,231	1	,135
		DEQ_4_korr(5)	,074	1	,785
		DEQ_4_korr(6)	4,511	1	,034
		DEQ_4_korr(7)	,690	1	,406
		DEQ_4_korr(8	3,740	1	,053

)				
	Gesamtstatistik	16,842	8	,032

```
Block 1: Methode = Einschluß
```

Omnibus-Tests der Modellkoeffizienten

		Chi-Quadrat	df	Sig.
Schritt 1	Schritt	19,026	8	,015
	Block	19,026	8	,015
	Modell	19,026	8	,015

Modellzusammenfassung

Schritt	-2 Log-Likelihood	Cox & Snell R-Quadrat	Nagelkerkes R-Quadrat
1	96,397[a]	,185	,260

a. Schätzung beendet bei Iteration Nummer 20 weil die Höchstzahl der Iterationen erreicht wurde. Endlösung kann nicht gefunden werden.

Klassifizierungstabelle[a]

			Vorhergesagt		
			SUD1_0		Prozentsatz der Richtigen
	Beobachtet		0	1	
Schritt 1	SUD1_0	0	61	3	95,3
		1	20	9	31,0
	Gesamtprozentsatz				75,3

a. Der Trennwert lautet ,500

Variablen in der Gleichung

	Regressionskoeffizient B	Standardfehler	Wald	df	Sig.

Schritt 1[a]	DEQ_4_korr			3,875	8	,868
	DEQ_4_korr(1)	-20,216	23205,422	,000	1	,999
	DEQ_4_korr(2)	-20,216	20096,485	,000	1	,999
	DEQ_4_korr(3)	22,190	40192,969	,000	1	1,000
	DEQ_4_korr(4)	22,190	40192,969	,000	1	1,000
	DEQ_4_korr(5)	-,111	1,186	,009	1	,925
	DEQ_4_korr(6)	22,190	28420,721	,000	1	,999
	DEQ_4_korr(7)	,987	1,035	,909	1	,340
	DEQ_4_korr(8)	2,086	1,186	3,096	1	,078
	Konstante	-,987	,269	13,496	1	<,001

Variablen in der Gleichung

			95% Konfidenzintervall für EXP(B)	
		Exp(B)	Unterer Wert	Oberer Wert
Schritt 1[a]	DEQ_4_korr			
	DEQ_4_korr(1)	,000	,000	.
	DEQ_4_korr(2)	,000	,000	.
	DEQ_4_korr(3)	4336274578,179	,000	.
	DEQ_4_korr(4)	4336274578,179	,000	.
	DEQ_4_korr(5)	,895	,088	9,138
	DEQ_4_korr(6)	4336274578,179	,000	.
	DEQ_4_korr(7)	2,684	,353	20,428
	DEQ_4_korr(8)	8,053	,788	82,242

Konstante	,373		

a. In Schritt 1 eingegebene Variablen: DEQ_4_korr.

Logistische Regression

Hinweise

Ausgabe erstellt		11-MAR-2026 18:19:51
Kommentare		
Eingabe	Daten	H:\Gabapentin Auswertung\28.02.24\6.1.2022 Gabapentin_1.sav
	Aktiver Datensatz	DataSet1
	Filter	<keine>
	Gewichtung	<keine>
	Aufgeteilte Datei	<keine>
	Anzahl der Zeilen in der Arbeitsdatei	93
Behandlung fehlender Werte	Definition für "fehlend"	Benutzerdefinierte fehlende Werte werden als fehlend behandelt
Syntax		LOGISTIC REGRESSION VARIABLES SUD1_0 /METHOD=ENTER DEQ_5_korr /CONTRAST (DEQ_5_korr)=Indicator(1) /PRINT=CI(95) /CRITERIA=PIN(0.05) POUT(0.10) ITERATE(20) CUT(0.5).

Ressourcen	Prozessorzeit	00:00:00,00
	Verstrichene Zeit	00:00:00,01

Zusammenfassung der Fallverarbeitung

Ungewichtete Fälle[a]		N	Prozent
Ausgewählte Fälle	Einbezogen in Analyse	93	100,0
	Fehlende Fälle	0	,0
	Gesamt	93	100,0
Nicht ausgewählte Fälle		0	,0
Gesamt		93	100,0

a. Wenn die Gewichtung wirksam ist, finden Sie die Gesamtzahl der Fälle in der Klassifizierungstabelle.

Codierung abhängiger Variablen

Ursprünglicher Wert	Interner Wert
0	0
1	1

Codierungen kategorialer Variablen

			Parametercodierung				
		Häufigkeit	(1)	(2)	(3)	(4)	(5)
DEQ_5_korr	1	13	,000	,000	,000	,000	,000
	2	5	1,000	,000	,000	,000	,000
	3	5	,000	1,000	,000	,000	,000
	5	4	,000	,000	1,000	,000	,000
	6	3	,000	,000	,000	1,000	,000
	7	3	,000	,000	,000	,000	1,000
	8	10	,000	,000	,000	,000	,000
	9	5	,000	,000	,000	,000	,000
	10	45	,000	,000	,000	,000	,000

Codierungen kategorialer Variablen

Parametercodierung		
(6)	(7)	(8)

DEQ_5_korr	1	,000	,000	,000
	2	,000	,000	,000
	3	,000	,000	,000
	5	,000	,000	,000
	6	,000	,000	,000
	7	,000	,000	,000
	8	1,000	,000	,000
	9	,000	1,000	,000
	10	,000	,000	1,000

Block 0: Anfangsblock

Klassifizierungstabelle[a,b]

			Vorhergesagt		
			SUD1_0		Prozentsatz der Richtigen
Beobachtet			0	1	
Schritt 0	SUD1_0	0	64	0	100,0
		1	29	0	,0
	Gesamtprozentsatz				68,8

a. Konstante in das Modell einbezogen.

b. Der Trennwert lautet ,500

Variablen in der Gleichung

		RegressionskoeffizientB	Standardfehler	Wald	df	Sig.	Exp(B)
Schritt 0	Konstante	-,792	,224	12,505	1	<,001	,453

Variablen nicht in der Gleichung

			Wert	df	Sig.
Schritt 0	Variablen	DEQ_5_korr	9,032	8	,340
		DEQ_5_korr(1)	,191	1	,662
		DEQ_5_korr(2)	2,394	1	,122

	DEQ_5_korr(3)	,690	1	,406
	DEQ_5_korr(4)	1,819	1	,177
	DEQ_5_korr(5)	1,405	1	,236
	DEQ_5_korr(6)	,406	1	,524
	DEQ_5_korr(7)	2,045	1	,153
	DEQ_5_korr(8)	,214	1	,644
Gesamtstatistik		9,032	8	,340

```
Block 1: Methode = Einschluß
```

Omnibus-Tests der Modellkoeffizienten

		Chi-Quadrat	df	Sig.
Schritt 1	Schritt	10,989	8	,202
	Block	10,989	8	,202
	Modell	10,989	8	,202

Modellzusammenfassung

Schritt	-2 Log-Likelihood	Cox & Snell R-Quadrat	Nagelkerkes R-Quadrat
1	104,434[a]	,111	,157

a. Schätzung beendet bei Iteration Nummer 20 weil die Höchstzahl der Iterationen erreicht wurde. Endlösung kann nicht gefunden werden.

Klassifizierungstabelle[a]

	Vorhergesagt		
	SUD1_0		Prozentsatz der Richtigen
Beobachtet	0	1	

Schritt 1	SUD1_0	0	59	5	92,2
		1	22	7	24,1
	Gesamtprozentsatz				71,0

a. Der Trennwert lautet ,500

Variablen in der Gleichung

		RegressionskoeffizientB	Standardfehler	Wald	df	Sig.
Schritt 1[a]	DEQ_5_korr			4,466	8	,813
	DEQ_5_korr(1)	,799	1,125	,503	1	,478
	DEQ_5_korr(2)	-19,999	17974,843	,000	1	,999
	DEQ_5_korr(3)	1,204	1,197	1,011	1	,315
	DEQ_5_korr(4)	1,897	1,390	1,862	1	,172
	DEQ_5_korr(5)	-19,999	23205,422	,000	1	,999
	DEQ_5_korr(6)	,799	,922	,750	1	,386
	DEQ_5_korr(7)	1,609	1,125	2,045	1	,153
	DEQ_5_korr(8)	,303	,736	,170	1	,680
	Konstante	-1,204	,658	3,345	1	,067

Variablen in der Gleichung

			95% Konfidenzintervall für EXP(B)	
		Exp(B)	Unterer Wert	Oberer Wert
Schritt 1[a]	DEQ_5_korr			
	DEQ_5_korr(1)	2,222	,245	20,174
	DEQ_5_korr(2)	,000	,000	.
	DEQ_5_korr(3	3,333	,319	34,830

	)			
	DEQ_5_korr(4)	6,667	,437	101,732
	DEQ_5_korr(5)	,000	,000	.
	DEQ_5_korr(6)	2,222	,365	13,538
	DEQ_5_korr(7)	5,000	,551	45,391
	DEQ_5_korr(8)	1,354	,320	5,729
	Konstante	,300		

a. In Schritt 1 eingegebene Variablen: DEQ_5_korr.

Logistische Regression

Hinweise

Ausgabe erstellt		11-MAR-2026 18:19:51
Kommentare		
Eingabe	Daten	H:\Gabapentin Auswertung\28.02.24\6.1.2022 Gabapentin_1.sav
	Aktiver Datensatz	DataSet1
	Filter	<keine>
	Gewichtung	<keine>
	Aufgeteilte Datei	<keine>
	Anzahl der Zeilen in der Arbeitsdatei	93
Behandlung fehlender Werte	Definition für "fehlend"	Benutzerdefinierte fehlende Werte werden als fehlend behandelt
Syntax		LOGISTIC REGRESSION

		VARIABLES SUD1_0 /METHOD=ENTER DEQ_6_korr /CONTRAST (DEQ_6_korr)=Indicator(1) /PRINT=CI(95) /CRITERIA=PIN(0.05) POUT(0.10) ITERATE(20) CUT(0.5).
Ressourcen	Prozessorzeit	00:00:00,02
	Verstrichene Zeit	00:00:00,01

Zusammenfassung der Fallverarbeitung

Ungewichtete Fälle[a]		N	Prozent
Ausgewählte Fälle	Einbezogen in Analyse	93	100,0
	Fehlende Fälle	0	,0
	Gesamt	93	100,0
Nicht ausgewählte Fälle		0	,0
Gesamt		93	100,0

a. Wenn die Gewichtung wirksam ist, finden Sie die Gesamtzahl der Fälle in der Klassifizierungstabelle.

Codierung abhängiger Variablen

Ursprünglicher Wert	Interner Wert
0	0
1	1

Codierungen kategorialer Variablen

			Parametercodierung				
		Häufigkeit	(1)	(2)	(3)	(4)	(5)
DEQ_6_korr	1	72	,000	,000	,000	,000	,000
	2	12	1,000	,000	,000	,000	,000
	3	2	,000	1,000	,000	,000	,000

	6	1	,000	,000	1,000	,000	,000
	7	1	,000	,000	,000	1,000	,000
	8	2	,000	,000	,000	,000	1,000
	10	3	,000	,000	,000	,000	,000

Codierungen kategorialer Variablen

		Parametercodierung
		(6)
DEQ_6_korr	1	,000
	2	,000
	3	,000
	6	,000
	7	,000
	8	,000
	10	1,000

```
Block 0: Anfangsblock
```

Klassifizierungstabelle[a,b]

			Vorhergesagt		
			SUD1_0		Prozentsatz der Richtigen
Beobachtet			0	1	
Schritt 0	SUD1_0	0	64	0	100,0
		1	29	0	,0
	Gesamtprozentsatz				68,8

a. Konstante in das Modell einbezogen.

b. Der Trennwert lautet ,500

Variablen in der Gleichung

	Regressionskoeffizient B	Standardfehler	Wald	df	Sig.	Exp(B)

Schritt 0	Konstante	-,792	,224	12,505	1	<,001	,453

Variablen nicht in der Gleichung

			Wert	df	Sig.
Schritt 0	Variablen	DEQ_6_korr	8,990	6	,174
		DEQ_6_korr(1)	,706	1	,401
		DEQ_6_korr(2)	,926	1	,336
		DEQ_6_korr(3)	,458	1	,499
		DEQ_6_korr(4)	2,231	1	,135
		DEQ_6_korr(5)	4,511	1	,034
		DEQ_6_korr(6)	,007	1	,935
	Gesamtstatistik		8,990	6	,174

Block 1: Methode = Einschluß

Omnibus-Tests der Modellkoeffizienten

		Chi-Quadrat	df	Sig.
Schritt 1	Schritt	10,222	6	,116
	Block	10,222	6	,116
	Modell	10,222	6	,116

Modellzusammenfassung

Schritt	-2 Log-Likelihood	Cox & Snell R-Quadrat	Nagelkerkes R-Quadrat
1	105,201[a]	,104	,146

a. Schätzung beendet bei Iteration Nummer 20 weil die Höchstzahl der Iterationen erreicht wurde. Endlösung kann nicht gefunden werden.

Klassifizierungstabelle[a]

Beobachtet			Vorhergesagt SUD1_0: 0	SUD1_0: 1	Prozentsatz der Richtigen
Schritt 1	SUD1_0	0	64	0	100,0
		1	26	3	10,3
	Gesamtprozentsatz				72,0

a. Der Trennwert lautet ,500

Variablen in der Gleichung

		RegressionskoeffizientB	Standardfehler	Wald	df	Sig.
Schritt 1[a]	DEQ_6_korr			,946	6	,988
	DEQ_6_korr(1)	,619	,642	,930	1	,335
	DEQ_6_korr(2)	-20,247	28420,722	,000	1	,999
	DEQ_6_korr(3)	-20,247	40192,970	,000	1	1,000
	DEQ_6_korr(4)	22,158	40192,969	,000	1	1,000
	DEQ_6_korr(5)	22,158	28420,721	,000	1	,999
	DEQ_6_korr(6)	,262	1,253	,044	1	,834
	Konstante	-,956	,263	13,188	1	<,001

Variablen in der Gleichung

		Exp(B)	95% Konfidenzintervall für EXP(B): Unterer Wert	Oberer Wert
Schritt 1[a]	DEQ_6_korr			
	DEQ_6_korr(1)	1,857	,528	6,535
	DEQ_6_korr(2)	,000	,000	.

	DEQ_6_korr(3)	,000	,000	.
	DEQ_6_korr(4)	4200234591,413	,000	.
	DEQ_6_korr(5)	4200234591,413	,000	.
	DEQ_6_korr(6)	1,300	,112	15,144
	Konstante	,385		

a. In Schritt 1 eingegebene Variablen: DEQ_6_korr.

```
Logistische Regression
```

Hinweise

Ausgabe erstellt		11-MAR-2026 18:19:51
Kommentare		
Eingabe	Daten	H:\Gabapentin Auswertung\28.02.24\6.1.2022 Gabapentin_1.sav
	Aktiver Datensatz	DataSet1
	Filter	<keine>
	Gewichtung	<keine>
	Aufgeteilte Datei	<keine>
	Anzahl der Zeilen in der Arbeitsdatei	93
Behandlung fehlender Werte	Definition für "fehlend"	Benutzerdefinierte fehlende Werte werden als fehlend behandelt
Syntax		LOGISTIC REGRESSION VARIABLES SUD1_0 /METHOD=ENTER Entspannung_korr

		/CONTRAST (Entspannung_korr)=Indicator(1) /PRINT=CI(95) /CRITERIA=PIN(0.05) POUT(0.10) ITERATE(20) CUT(0.5).
Ressourcen	Prozessorzeit	00:00:00,00
	Verstrichene Zeit	00:00:00,01

Zusammenfassung der Fallverarbeitung

Ungewichtete Fälle[a]		N	Prozent
Ausgewählte Fälle	Einbezogen in Analyse	93	100,0
	Fehlende Fälle	0	,0
	Gesamt	93	100,0
Nicht ausgewählte Fälle		0	,0
Gesamt		93	100,0

a. Wenn die Gewichtung wirksam ist, finden Sie die Gesamtzahl der Fälle in der Klassifizierungstabelle.

Codierung abhängiger Variablen

Ursprünglicher Wert	Interner Wert
0	0
1	1

Codierungen kategorialer Variablen

			Parametercodierung				
		Häufigkeit	(1)	(2)	(3)	(4)	(5)
Entspannung_korr	1	46	,000	,000	,000	,000	,000
	2	4	1,000	,000	,000	,000	,000
	3	4	,000	1,000	,000	,000	,000
	4	7	,000	,000	1,000	,000	,000
	5	2	,000	,000	,000	1,000	,000
	6	4	,000	,000	,000	,000	1,000

	7	4	,000	,000	,000	,000	,000
	8	4	,000	,000	,000	,000	,000
	9	6	,000	,000	,000	,000	,000
	10	12	,000	,000	,000	,000	,000

Codierungen kategorialer Variablen

		Parametercodierung			
		(6)	(7)	(8)	(9)
Entspannung_korr	1	,000	,000	,000	,000
	2	,000	,000	,000	,000
	3	,000	,000	,000	,000
	4	,000	,000	,000	,000
	5	,000	,000	,000	,000
	6	,000	,000	,000	,000
	7	1,000	,000	,000	,000
	8	,000	1,000	,000	,000
	9	,000	,000	1,000	,000
	10	,000	,000	,000	1,000

Block 0: Anfangsblock

Klassifizierungstabelle[a,b]

			Vorhergesagt		
			SUD1_0		Prozentsatz der Richtigen
	Beobachtet		0	1	
Schritt 0	SUD1_0	0	64	0	100,0
		1	29	0	,0
	Gesamtprozentsatz				68,8

a. Konstante in das Modell einbezogen.

b. Der Trennwert lautet ,500

Variablen in der Gleichung

	Regressionsk	Standardfehle	Wald	df	Sig.	Exp(B)

		oeffizientB	r				
Schritt 0	Konstante	-,792	,224	12,505	1	<,001	,453

Variablen nicht in der Gleichung

			Wert	df	Sig.
Schritt 0	Variablen	Entspannung_korr	9,506	9	,392
		Entspannung_korr(1)	,690	1	,406
		Entspannung_korr(2)	,690	1	,406
		Entspannung_korr(3)	,481	1	,488
		Entspannung_korr(4)	,337	1	,561
		Entspannung_korr(5)	1,894	1	,169
		Entspannung_korr(6)	1,894	1	,169
		Entspannung_korr(7)	,690	1	,406
		Entspannung_korr(8)	,014	1	,906
		Entspannung_korr(9)	2,273	1	,132
	Gesamtstatistik		9,506	9	,392

```
Block 1: Methode = Einschluß
```

Omnibus-Tests der Modellkoeffizienten

		Chi-Quadrat	df	Sig.
Schritt 1	Schritt	11,574	9	,238
	Block	11,574	9	,238
	Modell	11,574	9	,238

Modellzusammenfassung

Schritt	-2 Log-Likelihood	Cox & Snell R-Quadrat	Nagelkerkes R-Quadrat
1	103,849[a]	,117	,165

a. Schätzung beendet bei Iteration Nummer 20 weil die Höchstzahl der Iterationen erreicht wurde. Endlösung kann nicht gefunden werden.

Klassifizierungstabelle[a]

Beobachtet			Vorhergesagt		
			SUD1_0		Prozentsatz der Richtigen
			0	1	
Schritt 1	SUD1_0	0	51	13	79,7
		1	16	13	44,8
	Gesamtprozentsatz				68,8

a. Der Trennwert lautet ,500

Variablen in der Gleichung

		RegressionskoeffizientB	Standardfehler	Wald	df	Sig.
Schritt 1[a]	Entspannung_korr			5,113	9	,824
	Entspannung_korr(1)	1,157	1,058	1,197	1	,274
	Entspannung_korr(2)	1,157	1,058	1,197	1	,274
	Entspannung_korr(3)	,870	,838	1,076	1	,300
	Entspannung_korr(4)	1,157	1,456	,632	1	,427
	Entspannung_korr(5)	-20,045	20096,485	,000	1	,999
	Entspannung_korr(6)	-20,045	20096,485	,000	1	,999
	Entspannung_korr(7)	1,157	1,058	1,197	1	,274
	Entspannung_korr(8)	,464	,932	,248	1	,619
	Entspannung_korr(	1,157	,673	2,959	1	,085

	9)					
	Konstante	-1,157	,346	11,213	1	<,001

Variablen in der Gleichung

			95% Konfidenzintervall für EXP(B)	
		Exp(B)	Unterer Wert	Oberer Wert
Schritt 1[a]	Entspannung_korr			
	Entspannung_korr(1)	3,182	,400	25,310
	Entspannung_korr(2)	3,182	,400	25,310
	Entspannung_korr(3)	2,386	,461	12,341
	Entspannung_korr(4)	3,182	,183	55,194
	Entspannung_korr(5)	,000	,000	.
	Entspannung_korr(6)	,000	,000	.
	Entspannung_korr(7)	3,182	,400	25,310
	Entspannung_korr(8)	1,591	,256	9,894
	Entspannung_korr(9)	3,182	,851	11,898
	Konstante	,314		

a. In Schritt 1 eingegebene Variablen: Entspannung_korr.

Logistische Regression

Hinweise

Ausgabe erstellt		11-MAR-2026 18:19:51
Kommentare		
Eingabe	Daten	H:\Gabapentin Auswertung\28.02.24\6.1.2022 Gabapentin_1.sav
	Aktiver Datensatz	DataSet1
	Filter	<keine>
	Gewichtung	<keine>
	Aufgeteilte Datei	<keine>
	Anzahl der Zeilen in der Arbeitsdatei	93
Behandlung fehlender Werte	Definition für "fehlend"	Benutzerdefinierte fehlende Werte werden als fehlend behandelt
Syntax		LOGISTIC REGRESSION VARIABLES SUD1_0 /METHOD=ENTER Euphorie_korr /CONTRAST (Euphorie_korr)=Indicator(1) /PRINT=CI(95) /CRITERIA=PIN(0.05) POUT(0.10) ITERATE(20) CUT(0.5).
Ressourcen	Prozessorzeit	00:00:00,00
	Verstrichene Zeit	00:00:00,01

Zusammenfassung der Fallverarbeitung

Ungewichtete Fälle[a]		N	Prozent
Ausgewählte Fälle	Einbezogen in Analyse	93	100,0
	Fehlende Fälle	0	,0
	Gesamt	93	100,0
Nicht ausgewählte Fälle		0	,0
Gesamt		93	100,0

a. Wenn die Gewichtung wirksam ist, finden Sie die

Gesamtzahl der Fälle in der Klassifizierungstabelle.

Codierung abhängiger Variablen

Ursprünglicher Wert	Interner Wert
0	0
1	1

Codierungen kategorialer Variablen

			Parametercodierung				
		Häufigkeit	(1)	(2)	(3)	(4)	(5)
Euphorie_korr	1	72	,000	,000	,000	,000	,000
	2	10	1,000	,000	,000	,000	,000
	3	1	,000	1,000	,000	,000	,000
	4	4	,000	,000	1,000	,000	,000
	5	3	,000	,000	,000	1,000	,000
	9	2	,000	,000	,000	,000	1,000
	10	1	,000	,000	,000	,000	,000

Codierungen kategorialer Variablen

		Parametercodierung
		(6)
Euphorie_korr	1	,000
	2	,000
	3	,000
	4	,000
	5	,000
	9	,000
	10	1,000

Block 0: Anfangsblock

Klassifizierungstabelle[a,b]

Beobachtet			Vorhergesagt SUD1_0: 0	Vorhergesagt SUD1_0: 1	Prozentsatz der Richtigen
Schritt 0	SUD1_0	0	64	0	100,0
		1	29	0	,0
	Gesamtprozentsatz				68,8

a. Konstante in das Modell einbezogen.

b. Der Trennwert lautet ,500

Variablen in der Gleichung

		RegressionskoeffizientB	Standardfehler	Wald	df	Sig.	Exp(B)
Schritt 0	Konstante	-,792	,224	12,505	1	<,001	,453

Variablen nicht in der Gleichung

			Wert	df	Sig.
Schritt 0	Variablen	Euphorie_korr	5,547	6	,476
		Euphorie_korr(1)	,007	1	,932
		Euphorie_korr(2)	,458	1	,499
		Euphorie_korr(3)	1,894	1	,169
		Euphorie_korr(4)	,007	1	,935
		Euphorie_korr(5)	,926	1	,336
		Euphorie_korr(6)	2,231	1	,135
	Gesamtstatistik		5,547	6	,476

Block 1: Methode = Einschluß

Omnibus-Tests der Modellkoeffizienten

		Chi-Quadrat	df	Sig.
Schritt 1	Schritt	7,729	6	,259
	Block	7,729	6	,259
	Modell	7,729	6	,259

Modellzusammenfassung

Schritt	-2 Log-Likelihood	Cox & Snell R-Quadrat	Nagelkerkes R-Quadrat
1	107,694[a]	,080	,112

a. Schätzung beendet bei Iteration Nummer 20 weil die Höchstzahl der Iterationen erreicht wurde. Endlösung kann nicht gefunden werden.

Klassifizierungstabelle[a]

	Beobachtet		Vorhergesagt SUD1_0 0	Vorhergesagt SUD1_0 1	Prozentsatz der Richtigen
Schritt 1	SUD1_0	0	64	0	100,0
		1	28	1	3,4
	Gesamtprozentsatz				69,9

a. Der Trennwert lautet ,500

Variablen in der Gleichung

		RegressionskoeffizientB	Standardfehler	Wald	df	Sig.
Schritt 1[a]	Euphorie_korr			,044	6	1,000
	Euphorie_korr(1)	-,154	,734	,044	1	,834
	Euphorie_korr(2)	-20,510	40192,970	,000	1	1,000
	Euphorie_korr(3)	-20,510	20096,485	,000	1	,999
	Euphorie_korr(4)	,000	1,250	,000	1	1,000

	Euphorie_korr(5)	-20,510	28420,722	,000	1	,999
	Euphorie_korr(6)	21,896	40192,969	,000	1	1,000
	Konstante	-,693	,250	7,687	1	,006

Variablen in der Gleichung

		Exp(B)	95% Konfidenzintervall für EXP(B) Unterer Wert	Oberer Wert
Schritt 1[a]	Euphorie_korr			
	Euphorie_korr(1)	,857	,203	3,612
	Euphorie_korr(2)	,000	,000	.
	Euphorie_korr(3)	,000	,000	.
	Euphorie_korr(4)	1,000	,086	11,588
	Euphorie_korr(5)	,000	,000	.
	Euphorie_korr(6)	3230949685,702	,000	.
	Konstante	,500		

a. In Schritt 1 eingegebene Variablen: Euphorie_korr.

Logistische Regression

Hinweise

Ausgabe erstellt		11-MAR-2026 18:19:51
Kommentare		
Eingabe	Daten	H:\Gabapentin

		Auswertung\28.02.24\6.1.2022 Gabapentin_1.sav
	Aktiver Datensatz	DataSet1
	Filter	<keine>
	Gewichtung	<keine>
	Aufgeteilte Datei	<keine>
	Anzahl der Zeilen in der Arbeitsdatei	93
Behandlung fehlender Werte	Definition für "fehlend"	Benutzerdefinierte fehlende Werte werden als fehlend behandelt
Syntax		LOGISTIC REGRESSION VARIABLES SUD1_0 /METHOD=ENTER Angst_korr /CONTRAST (Angst_korr)=Indicator(1) /PRINT=CI(95) /CRITERIA=PIN(0.05) POUT(0.10) ITERATE(20) CUT(0.5).
Ressourcen	Prozessorzeit	00:00:00,02
	Verstrichene Zeit	00:00:00,01

Zusammenfassung der Fallverarbeitung

Ungewichtete Fälle[a]		N	Prozent
Ausgewählte Fälle	Einbezogen in Analyse	93	100,0
	Fehlende Fälle	0	,0
	Gesamt	93	100,0
Nicht ausgewählte Fälle		0	,0
Gesamt		93	100,0

a. Wenn die Gewichtung wirksam ist, finden Sie die Gesamtzahl der Fälle in der Klassifizierungstabelle.

Codierung abhängiger

Variablen

Ursprünglicher Wert	Interner Wert
0	0
1	1

Codierungen kategorialer Variablen

		Häufigkeit	Parametercodierung (1)	(2)	(3)	(4)	(5)
Angst_korr	1	67	,000	,000	,000	,000	,000
	2	6	1,000	,000	,000	,000	,000
	3	5	,000	1,000	,000	,000	,000
	4	2	,000	,000	1,000	,000	,000
	5	3	,000	,000	,000	1,000	,000
	6	2	,000	,000	,000	,000	1,000
	8	4	,000	,000	,000	,000	,000
	9	1	,000	,000	,000	,000	,000
	10	3	,000	,000	,000	,000	,000

Codierungen kategorialer Variablen

		Parametercodierung (6)	(7)	(8)
Angst_korr	1	,000	,000	,000
	2	,000	,000	,000
	3	,000	,000	,000
	4	,000	,000	,000
	5	,000	,000	,000
	6	,000	,000	,000
	8	1,000	,000	,000
	9	,000	1,000	,000
	10	,000	,000	1,000

Block 0: Anfangsblock

Klassifizierungstabelle[a,b]

			Vorhergesagt		
			SUD1_0		Prozentsatz der Richtigen
Beobachtet			0	1	
Schritt 0	SUD1_0	0	64	0	100,0
		1	29	0	,0
	Gesamtprozentsatz				68,8

a. Konstante in das Modell einbezogen.

b. Der Trennwert lautet ,500

Variablen in der Gleichung

		RegressionskoeffizientB	Standardfehler	Wald	df	Sig.	Exp(B)
Schritt 0	Konstante	-,792	,224	12,505	1	<,001	,453

Variablen nicht in der Gleichung

			Wert	df	Sig.
Schritt 0	Variablen	Angst_korr	6,423	8	,600
		Angst_korr(1)	,014	1	,906
		Angst_korr(2)	,308	1	,579
		Angst_korr(3)	,337	1	,561
		Angst_korr(4)	1,819	1	,177
		Angst_korr(5)	,926	1	,336
		Angst_korr(6)	,690	1	,406
		Angst_korr(7)	,458	1	,499
		Angst_korr(8)	1,819	1	,177
	Gesamtstatistik		6,423	8	,600

Block 1: Methode = Einschluß

Omnibus-Tests der Modellkoeffizienten

		Chi-Quadrat	df	Sig.
Schritt 1	Schritt	6,920	8	,545
	Block	6,920	8	,545
	Modell	6,920	8	,545

Modellzusammenfassung

Schritt	-2 Log-Likelihood	Cox & Snell R-Quadrat	Nagelkerkes R-Quadrat
1	108,503[a]	,072	,101

a. Schätzung beendet bei Iteration Nummer 20 weil die Höchstzahl der Iterationen erreicht wurde. Endlösung kann nicht gefunden werden.

Klassifizierungstabelle[a]

	Beobachtet		Vorhergesagt SUD1_0 0	Vorhergesagt SUD1_0 1	Prozentsatz der Richtigen
Schritt 1	SUD1_0	0	59	5	92,2
		1	22	7	24,1
	Gesamtprozentsatz				71,0

a. Der Trennwert lautet ,500

Variablen in der Gleichung

		RegressionskoeffizientB	Standardfehler	Wald	df	Sig.
Schritt 1[a]	Angst_korr			4,372	8	,822
	Angst_korr(1)	,234	,907	,066	1	,797
	Angst_korr(2	-,460	1,150	,160	1	,690

	)					
	Angst_korr(3)	,927	1,440	,414	1	,520
	Angst_korr(4)	1,620	1,254	1,668	1	,197
	Angst_korr(5)	-20,276	28420,722	,000	1	,999
	Angst_korr(6)	,927	1,036	,800	1	,371
	Angst_korr(7)	-20,276	40192,970	,000	1	1,000
	Angst_korr(8)	1,620	1,254	1,668	1	,197
	Konstante	-,927	,271	11,691	1	<,001

Variablen in der Gleichung

			95% Konfidenzintervall für EXP(B)	
		Exp(B)	Unterer Wert	Oberer Wert
Schritt 1[a]	Angst_korr			
	Angst_korr(1)	1,263	,213	7,480
	Angst_korr(2)	,632	,066	6,021
	Angst_korr(3)	2,526	,150	42,479
	Angst_korr(4)	5,053	,432	59,054
	Angst_korr(5)	,000	,000	.
	Angst_korr(6)	2,526	,332	19,249
	Angst_korr(7)	,000	,000	.
	Angst_korr(8)	5,053	,432	59,054
	Konstante	,396		
